# New Artificial Biomimetic Enzyme Analogues Based on Iron(II/III) Schiff Base Complexes: An Effect of (Benz)imidazole Organic Moieties on Phenoxazinone Synthase and DNA Recognition ‡

**DOI:** 10.3390/molecules24173173

**Published:** 2019-08-31

**Authors:** Aleksandra Bocian, Martyna Szymańska, Daria Brykczyńska, Maciej Kubicki, Monika Wałęsa-Chorab, Giovanni N. Roviello, Marta A. Fik-Jaskółka, Adam Gorczyński, Violetta Patroniak

**Affiliations:** 1 Faculty of Chemistry, Adam Mickiewicz University, Uniwersytetu Poznańskiego 8, 61-614 Poznań, Poland; 2 Institute of Biostructures and Bioimaging—CNR, via Mezzocannone 16, 80134 Napoli, Italy

**Keywords:** iron, Schiff base, DNA binding, 2-aminophenol, biomimetic activity

## Abstract

Elucidation of the structure and function of biomolecules provides us knowledge that can be transferred into the generation of new materials and eventually applications in e.g., catalysis or bioassays. The main problems, however, concern the complexity of the natural systems and their limited availability, which necessitates utilization of simple biomimetic analogues that are, to a certain degree, similar in terms of structure and thus behaviour. We have, therefore, devised a small library of six tridentate *N*-heterocyclic coordinating agents (**L^1^**–**L^6^**), which, upon complexation, form two groups of artificial, monometallic non-heme iron species. Utilization of iron(III) chloride leads to the formation of the 1:1 (Fe:**L_n_**) ‘open’ complexes, whereas iron(II) trifluoromethanosulfonate allows for the synthesis of 1:2 (M:**L_n_**) ‘closed’ systems. The structural differences between the individual complexes are a result of the information encoded within the metallic centre and the chosen counterion, whereas the organic scaffold influences the observed properties. Indeed, the number and nature of the external hydrogen bond donors coming from the presence of (benz)imidazole moieties in the ligand framework are responsible for the observed biological behaviour in terms of mimicking *phenoxazinone synthase* activity and interaction with DNA.

## 1. Introduction

Nature exhibits an astonishing ability to construct sophisticated molecular machineries to target selected, otherwise hardly approachable by synthetic chemists, molecular transformations [1]. Metalloenzymes were recognized as one class of such species, with judiciously chosen transition metal ions and enzyme active sites being able to facilitate various redox processes in a catalytic and selective manner [2]. Knowledge gained from studies of their structure and function is of prime importance that renders chemistry the hallmark of modern science [3]. Nevertheless, the natural systems’ complexity necessitates the use of artificially constructed biomimetic analogues for further advancement of this discipline [4]. This solution provides access to simple, readily accessible species that retain certain structural features of enzymes, and so their chemical behaviour and function may become mimicked.

From transition metal cations that constitute the redox-active part of enzymes like copper, manganese, or iron, the latter one is of particular importance due to its low biotoxicity, wide availability in nature and electronic states that favour binding of O_2_ [5,6]. In particular, one may discriminate heme [7] and non-heme [8] Fe-based enzymes, classification, and functions that depend on the structural framework of its active site. The seminal, most widely studied examples of the former group involve studies on the families of horseradish peroxidases [9] and cytochromes P450 [10]. The second group is a much broader class of molecular species, which can be divided in accordance to the number of iron centres [8,11]. Notable representative examples include the bimetallic methane monooxygenase (MMO), which catalyses oxidation of hydrocarbons [12] or the monometallic ‘2-His-1-carboxylate facial triad’ of relevance for various oxidative transformations [13].

These oxidations are a result of the reductive dioxygen activation, which are dependent on the structural features of the artificial biomimetic analogue, specifically the coordination environment around the metallic centre(s) and its redox characteristics [14]. It is thus possible to generate small groups of structurally similar coordinating agents, where one could incorporate the chosen structural motif into the ligands scaffold and evaluate its effect on the biological outcome. Schiff base ligands were recognized as very potent candidates for the construction of such libraries due to their robustness and stability, facile synthetic methodologies and rich complexation behaviour [15,16]. Specifically, iron(II/III) complexes were investigated as DNA binding agents [17,18,19] or as *phenoxazinone synthase* (PHS) artificial analogues [16,20,21,22,23,24,25]. Interestingly, in Nature it is the copper(II) ion that catalyses oxidation of o-aminophenols to phenoxazinones [26,27], which means that PHS-active iron systems could be used to gain additional insight into the oxidative biological mechanisms [20,28].

**Scheme 1:**
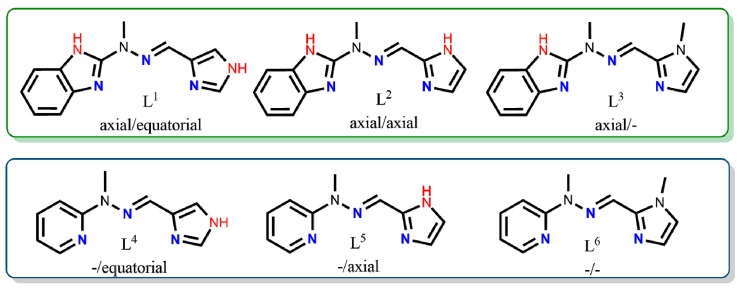
Schematic representation of ligands synthesized for the purpose of this work; green frame denotes the family of benzimidazole-scaffolded ligands whereas blue frame shows the family of pyridine-scaffolded coordinating agents.

Owing to our experience in the construction of the imine-scaffolded metallosupramolecular architectures [29,30,31,32,33,34,35,36,37] and bioassays [30,36,38,39,40], we designed and synthesized a small library of Schiff base ligands (Scheme 1), where the main structural difference involves the different number and disposition of the hydrogen bond donors.

Coordination with iron(II)/(III) metallic centres led to the formation of two families of complexes (‘open’ and ‘closed’ species) depending on the applied counterion, which were in turn investigated as artificial phenoxazinone synthase analogues and DNA-binding agents. To the best of our knowledge, this is the first report that studies the effect of H-bonding on the multifunctional behaviour of library of Schiff base iron agents in terms of bioassays.

## 2. Results and Discussion

### 2.1. Synthesis and Characterization

A series of six hydrazone Schiff base ligands **L^1^**–**L^6^** was successfully synthesized in a two-step synthetic protocol. [31] Commercially available 2-chlorobenzimidazole (in **L^1^**–**L^3^**) or 2-bromopyridine (in **L^4^**–**L^6^**) were subjected to nucleophilic substitution reaction with an excess of methylhydrazine and the product was reacted with the corresponding imidazolecarboxaldehyde. As a result, two sets of coordinating agents were obtained in 41.1–90% yields, with the common N_3_-tridentate meridional binding subunit which differed in the number (0–2) and topology (*axial* vs. *equatorial*) of donor hydrogen bonds (Scheme 1).

**Figure 1:**
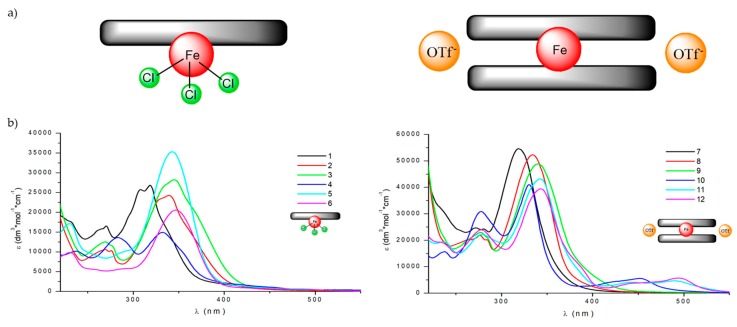
**a**) Schematic representation of compounds **A**: [Fe(**L^x^**)Cl_3_] ‘open’ (left) and **B:** [Fe(**L^x^**)_2_](OTf)_2_ ‘closed’ systems (right). **b**) UV-Vis spectra of ‘open’ **1**–**6** (left) and ‘closed’ system complexes **7**–**12** (right) recorded in MeOH at c = 2 × 10^−5^ M.

Their UV-Vis and NMR (when possible) solution studies (Figure 1b, Figures S11–S13) indicate stability of synthesized complexes in solution, indicating that the organic Schiff base ligands do not decoordinate. Interestingly, from the family of ‘closed’ species, the ones with the pyridine moiety **10**–**12** were found to be diamagnetic, whereas the benzimidazole-scaffolded ones **7**–**9** are paramagnetic at room temperature.

### 2.2. Description of Crystal Structures

The structures of iron complexes with six different ligands (**L^1^**–**L^6^**, cf. Scheme 1) were obtained. The crystal structures confirmed that these complexes can be formed either with one or two tridentate ligand molecules, therefore confirming our initial assumptions. Out of 12 possible compounds studied, we were able to determine crystal structures for nine complexes (cf. Experimental Section, Electronic Supplementary Information Figures S32–S35.

**Figure 2:**
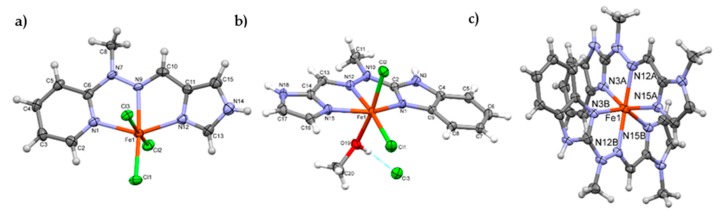
Representative perspective views of ‘open’ (**4**—**a**, **2**—**b**)) and ‘closed’ complexes (**9**—**c**); ellipsoids are drawn at the 50% probability level, hydrogen atoms are shown as spheres of arbitrary radii. Thin blue line in **2** represents the O–H···Cl^−^ hydrogen bond.

The first group consists of neutral complexes with the general formula [Fe(**L^x^**)Cl_3_] (with one exception of **2**, which is ionic and contains one coordinated methanol molecule instead of one of the chloride anions, additional Cl acts as a counterion). Figure 2 shows a representative example of these complexes.

In all these complexes Fe(III) cations are six coordinated, by three nitrogen atoms from ligand molecule and three Cl^−^ anions (in case of **2**, two Cl^−^ and methanol oxygen, Figure 2b) in a slightly distorted octahedral fashion. The distortion is mainly caused by *trans*-coordination of two nitrogen atoms of L^x^ molecule, appropriate N1-Fe-N15 angles differ significantly from 180°, they are in a range 147–148°. Table S1 lists the relevant geometrical features for all the complexes. In this group of complexes, the crystal structures are determined mainly by N-H···Cl (C-H···Cl) hydrogen bonds, and often by secondary π···π or C-H···π interactions (hydrogen bond data are listed in Table S2).

Second group contains cationic complexes of general formula [Fe(**L^x^**)_2_]^2+^, with two triflate anions balancing the charge. Also in these cases Fe(II) is six coordinated, by six nitrogen atoms from two ligand molecules, but distortions from ideal octahedral geometry are larger than in former group (cf. Table 1). Figure 2c shows example of the member of this group (**9**). In the crystal structures of this group the N-H···O (triflate) or C-H···O hydrogen bonds are always present. Probably due to more complicated packing of different moieties, in almost all examples the voids filled by solvent molecules. Table S1 contains also information of the size of the voids, determined as a part of the unit cell. All ligand molecules are almost or approximately planar, as may be seen by analyzing the dihedral angles between planar fragments of the molecules (Table S1).

In the crystal structures, Coulombic interactions between ions and directional N-H···O hydrogen bonds between cations and anions (Table S2) are important factors for final crystals architectures.

### 2.3. Catalysis and DNA Binding Affinity

The structural similarities present within each group of iron complexes represent a great starting point for determination of the structure/properties dependencies, specifically an influence of the number and disposition of H-bonding within the ligands framework on their biomimetic behavior as: (i) artificial phenoxazinone synthase analogues; (ii) DNA-recognition binders.

#### 2.3.1. Phenoxazinone Synthase (PHS) Activity

Oxidation of 2-aminophenol can be done using dioxygen or hydrogen peroxide [21], using different solvent as an environment [24] and what is the most important using a wide variety of complexes. Examples in the literature include: cobalt(II) [21,22,23], manganese(II) [21] or iron(III) compounds [20,28].

**Scheme 2:**
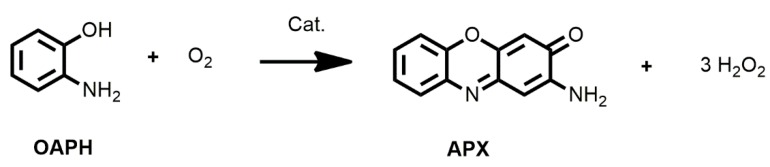
Schematic representation of oxidation of 2-aminophenol (OAPH) to 2-aminophenoxazine-3-one chromophore (APX).

At room temperature and in the presence of air, the present series of iron(II)/(III) complexes catalyze the aerobic oxidative dehydrogenation of o-aminophenol (OAPH) to the corresponding light-absorbing 2-aminophenoxazine-3-one chromophore (APX) (Scheme 2) and the process is strongly dependent on the chosen complex.

**Figure 3:**
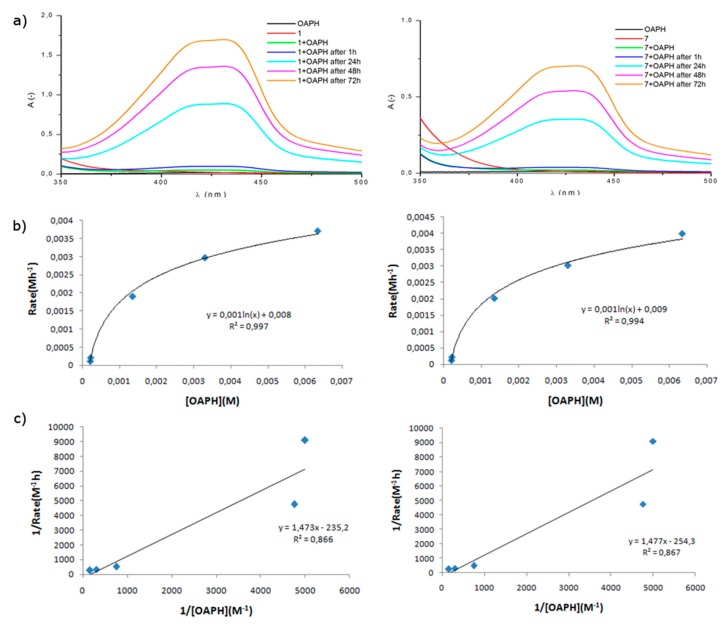
**a**) The spectral profile showing the growth of 2-aminophenoxazine-3-one at 433 nm due to addition of complex **1** (left) and **7** (right) to 2-aminophenol dissolved in methanol. The spectra were recorded under aerobic conditions during the three days. **b**) Plot of rate vs. concentration for complex **1** (left) and **7** (right). **c**) Linewear–Burk plot of phenoxazinone synthase-like activity for complex **1** (left) and **7** (right).

First of all, a ten-fold excess of o-aminophenol solution (2 × 10^−4^ M) was added to methanolic solutions of the chosen iron complex (2 × 10^−5^ M), so that an excess of substrate is present and first-order kinetic reaction mechanism could be maintained. The reaction was carried out at room temperature in the presence of air, without any base to minimize the possibility of auto-oxidation of OAPH [41] and in methanol for solubility issues. The catalytic properties of the complexes were monitored spectrophotometrically by observing the increasing intensity of the absorption band at 433 nm, which corresponds to the formation of APX chromophore. Measurements were made separately for the complex, *o*-aminophenol and also immediately after mixing of the substrates as a function of time. The chosen measurement time intervals (1 h, 24 h, 48 h, 72 h) are a result of minimal spectroscopic changes at first hours, most probably associated with high iron(III) oxidation state for ‘open’ complexes and sterically blocked ‘closed’ complexes. Spectra registered for complexes **1**–**12** are gathered in the Supplementary Information (Figures S37–S46) and the most catalytically active complexes from each of the two groups (**1** for ‘open’ and **7** for ‘closed’), are shown in Figure 3.

Thanks to observation of changes in the intensity of bands, it is possible to confirm the catalytic properties of the studied complexes, since blank tests without catalyst (Figure S36) confirm that the latter is necessary to obtain the observed conversion of OAPH to APX.

To gain deeper mechanistic insight into oxidation of OAPH with studied iron(II/III) complexes, kinetic studies were performed at room temperature for each system by varying the relative concentration of complexes from 2 × 10^−4^ M–2 × 10^−6^ M and measurement of the reaction initial rates at 433 nm. By ensuring an excess of the substrate with regard to the complex, the initial rates method exhibits a first-order dependence on complex concentration and was treated with the Michaelis–Menten model, in which linearization affords a double reciprocal Lineweaver–Burk plot allowing analysis of the following parameters: the maximum velocity (V_max_), binding constant (K_M_), and rate constant (k_cat_) (Figure 3b,c) and Figures S37–S46, Supplementary information). Indeed, rate saturation kinetics was observed, which confirms the validity of the applied kinetic model and also indicates that the reaction involves the formation of complex-substrate intermediate in a pre-equilibrium stage and its subsequent irreversible oxidation is associated as the rate-determining step of the catalytic cycle [25].

Table 1 includes all kinetic parameters determined for twelve iron complexes under study used in oxidation of OAPH, with the range of corresponding values being: V_max_ = 0.86–3.98 [10^−3^Ms^−1^], K_M_ = 1.45–2.08 [10^−3^ M] and k_cat_ = 42.76–199.40 [h^−1^].

Comparison of different catalysts can be done by k_cat_, which is also expressed as the turnover number and can be related to its activity in a given time unit. Table 2 compares our compounds with different ones found in the literature and shows that our systems can be regarded as functional models of phenoxazinone synthase (PHS) which is involved in the biosynthesis of Actinomycin D, the latter of which is a naturally occurring nontrivial APX derivative exhibiting anti-cancer properties [42,43,44]. We did observe however that activity of each complex is an interplay of its ‘primary’ (open vs. closed) and ‘secondary’ (number and disposition of H-bonds) structural features.

Supplementary Table S3, which helps in following the structural features as a function of catalytic parameters is provided in Supplementary Information. It must be noted that whereas higher k_cat_ denotes higher activity of a given complex, lower K_M_ values indicate stronger binding of complex to OAPH which is related to TON, yet might be limited by further reaction processes.

Within the group of ‘open’ complexes **1**–**6**, K_M_ decreases in the order of **L^6^** > ~**L^2^** > **L^5^** = **L^4^** > **L^3^** > **L^1^** and seems to be affected by the ligands H-bonding scaffold. Indeed, whereas the highest value is found for **L^6^** with no H-bond donors, ca. 10% decrease was observed for complexes with scaffold comprising one NH moiety. Interestingly, its relative disposition (**L^5^** vs. **L^4^**) does not seem to have relevant influence herein. Further decrease in the series exhibits iron(III) complex coordinated to benzimidazole-imidazole ligand **L^1^** of a mixed axial/equatorial H-bond disposition. Quite unexpectedly, isomeric ligand **L^2^** from iron complex interacts with OAPH in a similar fashion as **L^6^**, though their TON values differ. Since coordination number 6 of Fe(III) metal ion can be safely termed as saturated, its interaction with OAPH is possible by assuming exchange of at least one coordinated chloride anion with solvent molecules. Indeed, X-ray structure that we provide for complex **2** proves that it was possible to isolate such species in the solid state as [Fe(**L^2^**)Cl_2_(MeOH)]Cl. Consequently, OAPH can be assumed to coordinate to iron(III) via phenoxo anion, with concomitant exchange of coordinated methanol molecule. This is where non-binding NH_2_ moiety from coordinated OAP group could additionally interact with NH units of ligand scaffold. (compare with proposed mechanism in Section 2.3.2). TONs change within the order of **L^1^** > **L^3^** > **L^6^** > **L^4^**~**L^2^** > **L^5^** and seem to not be dependent on H-bonding moieties (specifically compare K_M_ and TONs of **4** and **6**), which also prove that the rate-determining step would be associated with irreversible oxidation of the OAP-catalyst complex, not the binding of OAPH with catalyst per se.

Interesting comparison comes with studies of the ‘closed’ complexes. One could expect that they would not be catalytically active, since it should be hard to dissociate one of the tridentate imine ligands. In addition, ^1^HNMR studies of diamagnetic **10**–**12** complexes do not show formation of multiple species in solution. It turns out that TONs decrease in the following order **L^1^** > **L^3^** > **L^6^** > **L^2^**~**L^5^** > **L^4^**, with the most active complexes being ones based on **L^1^** and **L^3^** ligands – similarly as in the ‘open’ class. Intriguingly, K_M_ decreases in exactly the same order**,** which would imply that appropriate number and disposition of H-bonding between OAPH and coordinated ligand should be responsible for generation of OAPH···complex interactions. NH on imidazole moiety seems to influence such an interaction more significantly than the benzimidazole unit, however since TONs and K_M_ follow the same trend, redox Fe(II/III) oxidation changes would explain oxidation of OAPH in spite of binding of the latter one via H—bonding and not cationic centre. All in all, ‘closed’ species are somewhat less efficient for oxidation of OAPH than ‘open’ species.

#### 2.3.2. Proposed Mechanism of OAPH Oxidation

**Scheme 3:**
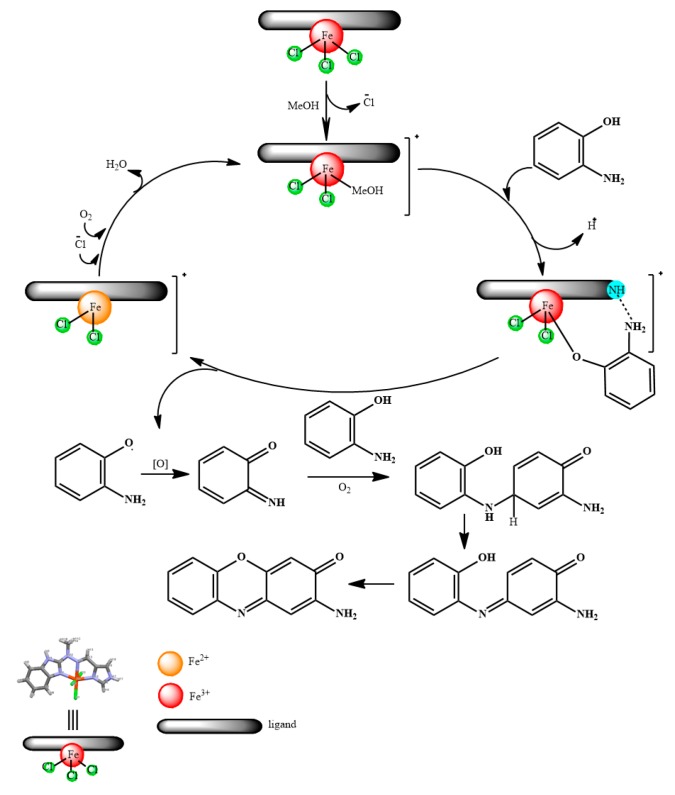
Proposed mechanism of phenoxazinone synthase like activity of complex **1** in methanolic environment of reaction.

On the basis of obtained results and literature data [25,41], we would like to show proposed mechanism of oxidation of 2-aminophenol (OAPH) to 2-aminophenoxazine-3-one (APX) with ‘open’ iron complexes investigated herein (Scheme 3).

As was noted earlier, ‘open’ species are prone to ligand exchange and this is what happens in the first step between chloride and methanol molecule. Such a configuration allows the molecule of complex to interact with molecule of 2-aminophenol (OAPH), which results in coordination of the latter one to the metallic centre in its deprotonated form, with simultaneous extrusion of the methanol solvent molecule to the environment and additional interactions from the ligand/H-bond moieties. Subsequent oxidation is presumed to be the rate-determining step, which could explain certain time-lag associated with the initial reaction times. Obtained 2-aminophenoxazinone (APX) is formed on the basis of quinone imine intermediate, reacting with second molecule of OAPH and a series of subsequent redox transformations [25,41].

#### 2.3.3. DNA Binding Affinity

The compounds may interact with DNA in a variety of ways: they can combine by electrostatic interaction with a phosphate sugar backbone, intercalate between pairs of bases, covalently bind with nucleobases or attach in major or minor grooves [48]. Binding ability of metal complexes to DNA can be studied by using spectroscopic methods such as: electronic absorption titration [49], fluorescence competitive binding with ethidium bromide (EB) [50] and circular dichroism (CD) measurements [51]. Based on the observed changes in the spectrum the type of interaction may be specified.

##### Absorption Titration

The absorption titration experiment provides basic information concerning the presence and strength of interactions between compounds and DNA. The most important and straightforward indication is hypochromism in the spectrum of compound that increases together with the increase of concentration of CT-DNA in the sample solution [52,53]. Monitoring of this phenomenon allows one to evaluate the ability of compounds to interact with DNA double helix.

**Figure 4:**
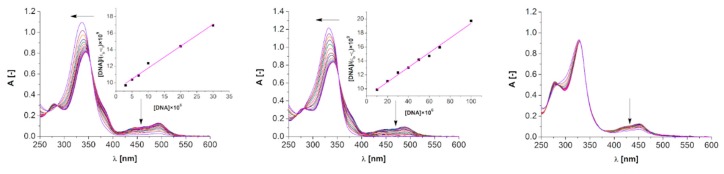
Absorption titration of **12** (left), **11** (middle) and **10** (right) with increasing concentrations of CT-DNA (0–100 µM). Arrows show hypsochromic and hypochromic changes upon increasing CT-DNA concentration. Inset: plot of [DNA]/(εa − εf) versus [DNA]; ▪,experimental data points; solid line, linear fitting of the data.

Absorption spectra of all complexes in the presence of CT-DNA were measured in the range of 250–600 nm (Figures S47–S49). Interestingly, neither of ‘open’ complexes interact with CT-DNA, and only three ‘closed’ complexes did show spectral changes attributable to specific complex-nucleic acid interactions (**10**, **11**, **12**—Figure 4). It shows the significance of the “secondary” structure of potential metallodrugs (if we say that the ligand framework and metal salt are the “primary” structure, then their mutual arrangement in the space may be called the “secondary” structure of the system). Interestingly, **10**–**12** employ pyridine moiety in the ligands “primary” structure, which would imply that introduction of additional H-bonding through benzimidazole moiety hampers such process. However, not only the pyridine/benzimidazole bias in the ligand scaffold is visible, since the topology and substitution pattern of imidazole is relevant for DNA binding as well. Compounds **11** and **12** with 2-imidazole substituent interact with DNA much stronger (hypochromism of MLCT band at ca. 485 nm of 96% and 79%, respectively) than **10** with 4-imidazole substituent (hypochromism of MLCT band of 45%). At the same time blocking of hydrogen bonding donor site N-H as N-CH_3_ in imidazole unit facilitates the DNA binding (comparison of **L^5^** and **L^6^** in complexes **11** and **12**). One may say that two-level recognition in the presented case is observed and is crucial for the DNA binding phenomenon: (i) lack of hydrogen bonding donors in the “primary” structure and (ii) ‘closed’ composition of the “secondary” structure of complex.

As mentioned before, interaction of complexes **10**, **11** and **12** with DNA is visible as a decrease of the intensity of the MLCT band at ca. λ = 485 nm. In general, a strong hypochromism in the absorption spectra is an indication of intercalative binding mode, since the distance between intercalated compound and DNA bases decreases and the π electrons of both combine [54]. Only for **11** and **12** the binding constants (K_b_) were calculated, since the diminution of the MLCT band in case of **10** did not reach 50%. In the face of this fact, we focused in our further discussion on **11** and **12**. Both complexes **11** and **12**, as well as the corresponding ligands (**L^5^** and **L^6^**), are stable in the biological medium employed in this study (10 mM TrisHCl, 5 mM NaCl, 50 mM pH 7.5) as shown in Figure S50. In general, **12** binds to the DNA twice more efficiently than **11** what is reflected in the intrinsic binding constants K_b_ = 2.8797 × 10^4^ (R^2^ = 0.98602 for 6 points, inset Figure 4) and K_b_ = 1.1820 × 10^4^ (R^2^ = 0.99087 for 8 points, inset Figure 4), for **12** and **11**, respectively. In case of both complexes, a hypsochromic shifts of ca. 8 nm of ILCT bands (ca. 335 nm) were observed. Changes in the intensity and position of the ligand-derived bands may suggest electrostatic interaction with CT-DNA. On the other hand, there is an isobestic point at 353 nm for complex **11** and 354 nm for complex **12** as well as hypochromism of MLCT bands at 488 nm and 493 nm for complexes **11** and **12**, respectively, indicating intercalative binding to DNA [15,55]. The standard Gibb’s free energy for DNA binding is −25.03 $\frac{kJ}{mol}$ and −22.86 $\frac{kJ}{mol}$ for **12** and **11**, respectively, which indicates the spontaneous binding of both compounds with DNA. Also, the ability of ligands **L^5^** and **L^6^** to bind with CT-DNA was examined (Figure S51). Since no significant changes in the intensity of bands as increasing amounts of DNA were added, one may assume that they do not interact with DNA in non-coordinated form.

##### Competitive Binding Fluorescence Experiment

In order to further investigate the strength of intercalation mode of synthesized complexes with DNA the competitive binding experiments with ethidium bromide (EB) as a probe were carried out. EB is a weak luminescent compound exhibiting high affinity to the DNA helix. Formation of a DNA–EB complex enhances its emission at λ_max_ = 590 nm (λ_exc_ = 467 nm) due to strong intercalation of EB planar phenanthridine rings between adjacent base pairs of the DNA helix. Displacement of EB from its DNA–EB complex due to gradual titration by a competing molecule results in subsequent quenching of its emission band.

**Figure 5:**
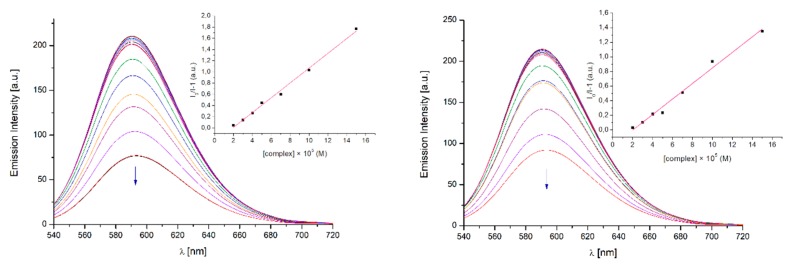
Emission spectra of ethidium bromide (EB) bound to CT DNA in the presence of increasing amount of **12** (left) and **11** (right). Arrow shows the hypochromic changes upon increasing **12** or **11** concentration. Inset: Stern–Volmer plot of I_0_/I − 1 versus [12] or [11] for the titration of EB-CT DNA complex; ▪, experimental data points; solid line, linear fitting of the data.

Competitive binding experiments with DNA-EB complex showed that compounds **11** and **12** are able to bind with the DNA scaffold and release EB from its complex. Significant decrease in emission intensity was observed for both complexes, which indicates that complexes bind with DNA in spaces occupied by EB or EB is released due to conformational changes in the double helix. The quenching constants are: K_SV_ = 1.32 × 10^4^ and K_SV_ = 1.06 × 10^4^ for **12** and **11** (Figure 5), respectively. Based on quenching constants, it can be concluded that complex **12** is slightly better binder than complex **11**, which is consistent with the results obtained in the electronic absorption titration experiments. This could be explained by the fact that lack of hydrogen bonds in the “primary” structure of **12** results in easier insertion between the base pairs of the DNA helix than **11**. Both complexes act on the principle of intercalation with DNA due to the “secondary” ’closed’ structure.

##### DNA Binding Investigation via CD

**Figure 6:**
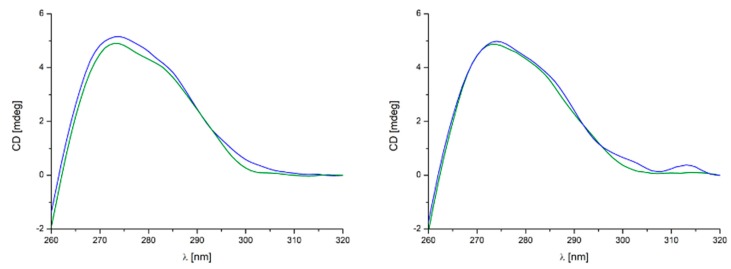
CD spectra relative to DNA duplex (2 µM) in the presence of 30 equiv. of **12** (**left**) and **11** (**right**) in Tris HCl 5 mM NaCl 50 mM (pH 7.5) at 20 °C (blue line) and after thermal treatment at 70 °C (5 min.) and subsequent cooling to 20 °C over 1 h in the same buffer (green line).

The study of morphological changes of DNA structure caused by the interaction of DNA with compounds can be observed using circular dichroism (CD) measurements. This technique is a very sensitive and informative method for studying the structural changes of the secondary structure of nucleic acids and proteins. The B-DNA helix exhibits two conservative bands due to its helicity—negative one at ca. 250 nm and a positive one at ca. 270 nm [50]. CD experiments were performed on the 12-mer oligo DNA of composition d(GTTAATCGCTGG) in the presence of different amounts of compounds **11** and **12** in the 1–30 eq. range. No significant CD perturbation under the above conditions was observed. However, after thermal treatment (70 °C) and subsequent slow cooling down of the sample solution, DNA underwent conformational changes in the presence of **12** corresponding to the variations observed in the positive band at 270 nm and are more pronounced for this ‘hydrogen bond free’ complex than in the case of **11**. Based on CD studies one may hypothesize that **12** is more effective than **11** (Figure 6) in interacting with DNA by preventing its correct annealing. Such findings from CD spectra correspond well with the results from DNA titration and EB competitive binding. Moreover, the differences observed in the CD DNA spectrum due to **12,** and in particular, the decrease of intensity of the positive band at 270 nm, resemble similar effects previously associated to well-characterized ligand-induced DNA conformational perturbations [16].

## 3. Experimental Section

### 3.1. Materials and Methods

All reagent and solvent were purchased form commercial sources and used as received.

Elemental analyses for C, H, and N were carried out using an Elementar Analyser Vario EL III (Elementar Analysensysteme GmbH, Hanau, Germany). Infrared spectra were recorded on an iS50 FT-IR, ThermoScientific Nicolet (Thermo Fisher Scientific wissenschaftliche Geräte GmbH, Wien, Austria). Electrospray ionisation mass spectroscopy (ESI-MS) was performed using Mass Spectrometer ZQ Waters (Miromass&Waters; Milford, USA) by dissolving the powder samples of compounds in methanol at ~10^−4^ M. The cone voltage values are depicted in each spectrum in Electronic Supplementary Material. ^1^H and ^13^C-NMR spectra were run on a Bruker Ultrashield 300 MHz spectrometer (Bruker, Karlsruhe, Germany) or Varian Gemini 400 MHz spectrometer (L^1^) (Varian, Darmstadt, Germany) and were calibrated against the residual protonated solvent signals with chemical shifts represented in ppm ((CD_3_)_2_SO: 2.50 ppm, water: 3.33 ppm; CD_3_CN: 1.94 ppm, water: 2.13 ppm; CDCl_3_: 7.26 ppm, water: 1.56 ppm; CD_3_OD: 3.31 ppm, water: 4.87 ppm). Each time ca. 2 mg of sample was dissolved in 0.6 mL of deuterated solvent.

CT-DNA, ethidium bromide, Tris and NaCl were supplied from Sigma Aldrich (Sigma Aldrich, Poznań, Poland) and used without further purification. CT-DNA was dissolved in Tris Buffer (5 mM TrisHCl, 50 mM NaCl, pH 7.25) prior to use. The CT-DNA solution gave a ratio of UV absorbance of 1.82:1 at 260 and 280 nm, indicating that the CT-DNA sample was sufficiently free from protein [56,57]. CT-DNA concentration per nucleotide was determined from the UV absorbance at 260 nm using the extinction coefficient ε_260_= 6600 dm^3^·mol^−1^·cm^−1^ [58]. Oligo DNA was supplied from Genomed S.A. (Genomed S.A., Warsaw, Poland) and used without further purification. Electronic absorption titrations of compounds were performed on Jasco V-770 spectrophotometer (Jasco Europe S.R.L., Cremella, Italy) equipped with a Peltier at a temperature of 20 °C between 600 and 250 nm, in 10 × 10 mm quartz cells. Emission spectra in the competitive fluorescence titration experiments were measured at room temperature on a JASCO FP-6200 spectrofluorimeter (Jasco, Tokyo, Japan) in the range 540–720 nm with excitation and emission slits of 10 nm and excitation wavelength λ_exc_ = 467 nm. CD experiments were performed by a Jasco J-810 spectropolarimeter (Jasco Europe S.R.L., Cremella, Italy) equipped with a Peltier PTC-423S/15 (Jasco Europe S.R.L., Cremella, Italy) with a response time of 4 s, a scanning speed of 50 nm/min and a bandwidth of 1.0 nm. Freshly prepared stock solutions of complexes (at concentration 2 × 10^−3^ M) were taken for all spectroscopic investigations of the binding mode with DNA. It needs to be emphasized that compounds are stable in this medium for several days. After this time some precipitate starts to occur.

### 3.2. Synthesis of Ligands

#### 3.2.1. Synthesis of Ligands **L^1^**–**L^3^**

**Scheme 4:**
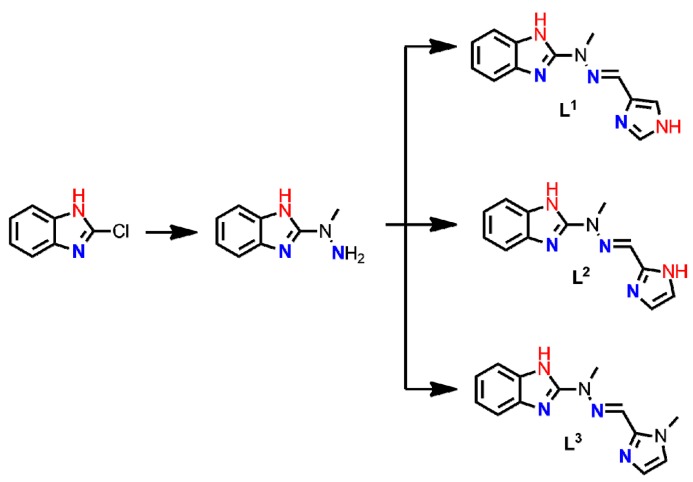
Schematic representation of ligands **L^1^**–**L^3^**.

**Scheme 5:**
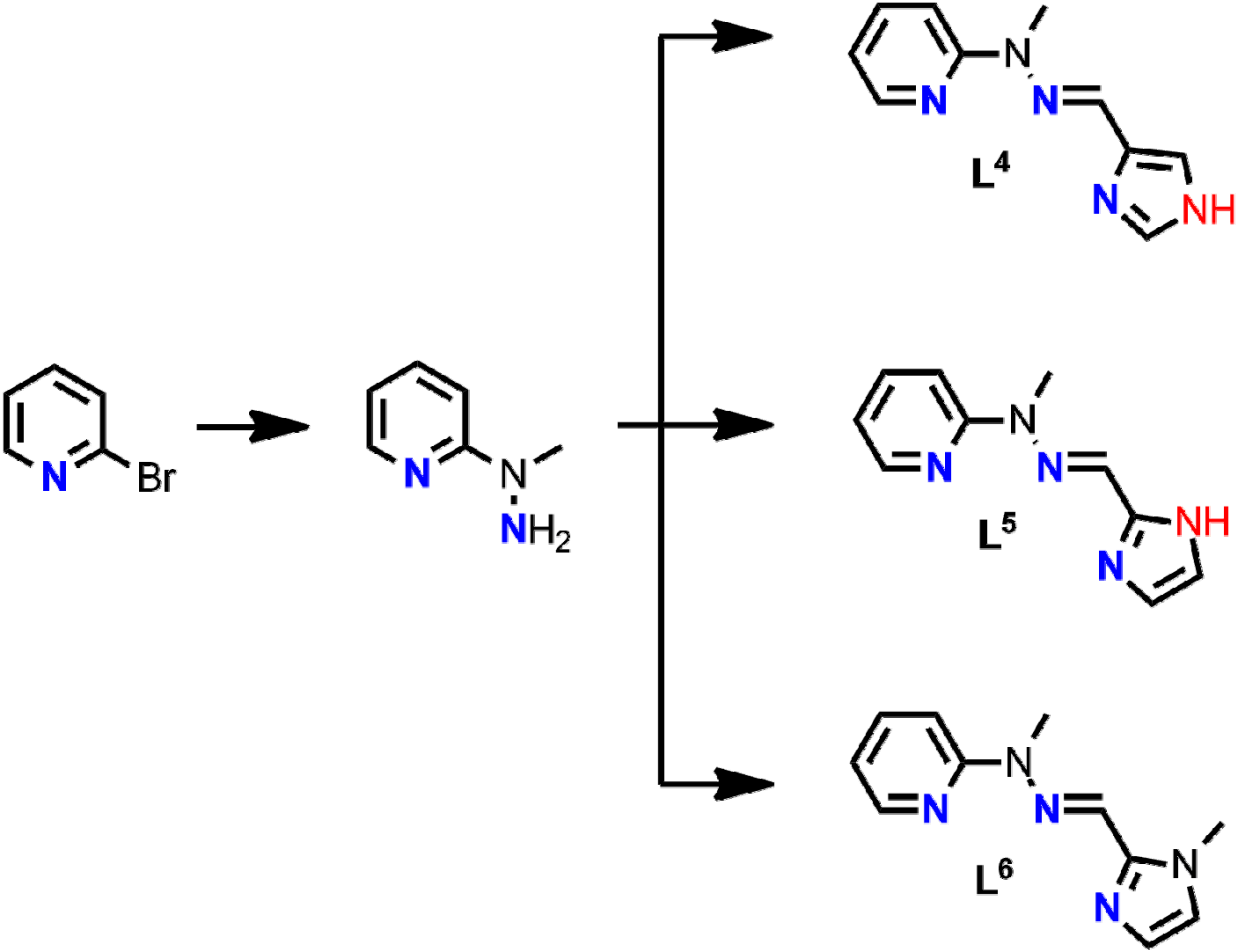
Schematic representation of ligands **L^4^**–**L^6^**.

Synthesized ligands (Scheme 4 and Scheme 5) were obtained as yellow, crystalline solids and their identity and purity were confirmed by routine analytical methods (^1^H-NMR, ^13^C-NMR, melting points, ESI-MS, Figures S1–S10, S14–S19). These species were subsequently reacted in equimolar ratio with iron(III) chloride and 2:1 (**L^x^**:M) iron(II) trifluoromethanesulfonate salts, respectively to obtain two classes of iron-based coordination compounds: **A**: [Fe(**L^x^**)Cl_3_] ‘open’ and **B**: [Fe(**L^x^**)_2_](OTf)_2_ ‘closed’ systems (Figure 1a). In general, weakly coordinating triflates do not form coordination bonds with Fe(II) ions, therefore two, tridentate Schiff base ligands fill its coordination sphere to form octahedrally coordinated ‘closed’ species. In the presence of chlorides the octahedral binding mode is preserved, nonetheless Fe(III) ions are bound by only one chelate imine ligand; the remaining coordination sites are potentially labile and are filled by three chlorides, hence ‘open’ species. Names refer to the coordination sites of the central metal ion, since chlorides are in fact more susceptible to exchange in the solvent medium than the chelating Schiff base ligand, which is also readily visible upon comparison of X-ray structures (e.g., **2** vs. **4**). We managed to successfully synthesize and characterize all 12 complexes (Figures S20–S31), 9 of which also by means of X-ray crystallography, and confirmed their structural character within each group, that is amenable to both the oxidation state of iron and the coordinative strength of the anion chosen during the synthesis. Details are included in Section 3.

The first step:

At two-necked round-bottomed flask 2-chlorobenzimidazole (8.00 g, 0.05 mol) was weighed and placed under argon atmosphere. Methylhydrazine (11.9 g, 0.25 mol) in five-fold excess was dissolved in a anhydrous ethanol and was added to the reaction mixture, which was warmed to 80 °C and stirred for 2 h. The white, crystalline product was filtered on Büchner funnel and dried under vacuum. Yield 68.2% (5.8 g, 0.036 mol).

The next step:

- for **L^1^**: condensation of 4-imidazolecarboxyaldehyde with 2-(1-methyl-hydrazine)-benzimidazole was performed. At two-necked round-bottomed flask 2-(1- methylhydrazine)benzimidazole (1.00 g, 6.16 mmol) was placed under argon atmosphere. The 4-imidazolecarboxyaldehyde (0.591 g, 6.16 mmol) was dissolved in a anhydrous ethanol and was added to the reaction mixture. Yellow suspension was formed and the reaction mixture was stirred for 24 h at 60 °C. Yellow clear solution was cooled to room temperature and the precipitate appeared, which was filtered by vacuum filtration, washed with anhydrous ethanol and dried under vacuum. Yield 80% (1.184 g, 4.9 mmol.)

ESI-MS(+) *m/z* (%): 241 (100) [H**L^1^**]^+^, 263 (10) [Na**L^1^**]^+^; ESI-MS(−): 239 (100) [**L^1^** − H]^−^. IR: ν(N-H)_br_ 3300–2500; ν(C=C)_ar_ 1596, 1560; ν(C-N) 1451; ν(C=N)_ar_ 1277; ρ(C-H)_ar._ 1162, 1091; γ(C-H)_ar._ 942, 737, 623 cm^−1^. ^1^H-NMR ((CD_3_)_2_SO, 300 MHz): δ (ppm) 12.38 (s, 0.7H, NH_(i/j)_); 11.53 (s, 0.7H, NH_(i/j)_); 7.89 (s, 1H, H_h_); 7.78 (s, 1H, H_f_); 7.31 (s, 3H, H_b,c,g_); 7.01 (s, 2H, H_a,d_); 4.37 (s, 0.7H, NH_(i/j)_); 3.56 (s, Me_(e)_). ^1^H-NMR ((CD_3_)_2_SO + K_2_CO_3_, 400 MHz): δ (ppm) 7.80 (s, 1H, H_h_); 7.77 (s, 1H, H_f_); 7.38 (s, 1H, H_g_); 7.32–7.28 (dd, 2H, *J* = 5.8, 3.2 Hz, H_b, c_); 7.00–6.96 (dd, 2H, *J* = 5.9, 3.2 Hz, H_a,d_); 3.56 (s, Me_(e)_). ^13^C-NMR ((CD_3_)_2_SO + K_2_CO_3_, 75 MHz): δ (ppm) 154.5, 138.7, 137.3, 131.9 (2C), 128.9, 125.2, 119.9 (2C), 112.7 (2C), 31.1. Melting temperature: 176–179 °C.

- for **L^2^**: condensation of 2-imidazolecarboxyaldehyde with 2-(1-methyl-hydrazine)-benzimidazole was performed. At two-necked round-bottomed flask 2-(1- methylhydrazine)benzimidazole (1.00 g, 6.16 mmol)was weighed and placed under argon atmosphere. 2-Imidazolecarboxyaldehyde (0.591 g, 6.16 mmol) was dissolved in anhydrous ethanol and was added to the reaction mixture. Yellow suspension was formed, and the reaction mixture was stirred for 24 h at 60 °C. Yellow clear solution was cooled to room temperature and evaporated to dryness. The resulting solid was dissolved in methanol and boiling acetonitrile was added to the flask. Cooling of the reaction mixture resulted in formation of pale pink crystalline product. The precipitate was filtered by vacuum filtration, washed with anhydrous ethanol and dried under vacuum to give 1.24 g (5.16 mmol) of ligand. The supernatant was concentrated to minimal amount of volume and the next part of precipitate was obtained 0.140 g (0.58 mmol). Total yield is 91% (1.38 g, 5.74 mmol).

ESI-MS(+) *m/z* (%): 241 (40) [H**L**^2^]^+^, 263 (100) [Na**L**^2^]^+^; ESI-MS(−): 239 (100) [**L^2^** − H]^−^. IR: ν(N-H)_br_ 3400–2400; ν(C=C)_ar._ 1552; ν(C-N) 1452; ν(C=N)_ar_ 1271; ρ(C-H)_ar._ 1145, 1040; γ(C-H)_ar._ 945, 737 cm^−1^. ^1^H-NMR ((CD_3_)_2_SO, 300 MHz): δ (ppm) 12.33 (s, 1H, NH_(i)_); 11.66 (s, 1H, NH_(j)_); 7.70 (s, 1H, H_f_); 7.45–7.28 (m, 3H, H_b, c, g_); 7.10–6.99 (m, 3H, H_a, d, h_); 3.59 (s, Me_(e)_). ^13^C-NMR ((CD_3_)_2_SO, 75 MHz): δ (ppm) 154.2, 144.9, 143.3, 134.1, 129.8, 127.8, 121.2, 120.4, 118.2, 116.9, 109.7, 31.7. Melting temperature: 295–306 °C. At 295 °C ligand started to become darker and decomposed at 306 °C.

- for **L^3^**: condensation of 1-methyl-2-imidazolecarboxyaldehyde with 2-(1-methyl-hydrazine)-benzimidazole was performed. At two-necked round-bottomed flask 2-(1- methylhydrazine)benzimidazole (1.00 g, 6.16 mmol)was weighed and placed under argon atmosphere. The 1-methyl-2-imidazolecarboxyaldehyde (0.704 g, 6.16 mmol) was dissolved in anhydrous ethanol and was added to the reaction mixture. Yellow suspension was formed and the reaction mixture was stirred for 24 h at 60 °C. Clear yellow solution was cooled to room temperature and kept in fridge. Yellow crystals were filtered by vacuum filtration, washed with anhydrous ethanol and dried under vacuum. Yield is 80% (1.26 g, 4.96 mmol).

ESI-MS(+) *m/z* (%): 255 (30) [H**L^3^**]^+^, 277 (100) [Na**L^3^**]^+^; ESI-MS(−): 253 (100) [**L^3^** − H]^−^. IR: ν(N-H)_br_ 3500-2450; ν(C=C)_ar._ 1573; ν(C-N) 1445; ν(C=N)_ar_ 1284; ρ(C-H)_ar._ 1170, 1038; γ(C-H)_ar._ 944, 743 cm^−1^. ^1^H-NMR ((CD_3_)_2_SO, 300 MHz): δ (ppm) 11.25 (s, 1H, H_(j)_), 7.81 (s, 1H, H_(f)_), 7.36–7.29 (m, 3H, H_b, c, h_), 7.04–6.99 (m, 3H, H_a, d, i_), 4.01 (s, Me_(g)_), 3.65 (s, Me_(e)_). ^13^C-NMR ((CD_3_)_2_SO, 75 MHz): δ (ppm) 153.4, 142.6, 144.1, 134.1, 129.8, 128.4, 124.3, 120.8, 119.8, 116.2, 110.1, 35.1, 31.6.

#### 3.2.2. Synthesis of Ligands **L^4^**–**L^6^**

The first step:

At two-necked round-bottomed flask 2-bromopyrridine (8.00 g, 0.05 mmol) was weighed and placed under argon atmosphere. Methylhydrazine (11.6 g, 0.25 mmol) was dissolved in anhydrous ethanol and was added to the reaction mixture. Then the reaction mixture was warmed to 80 °C and stirred for 24 h. The oil residue was purified by column chromatography (SiO_2_; 5% MeOH in CH_2_Cl_2_). Yield 52% (3.24 g).

The next step:

- for **L^4^**: condensation of 4-imidazolecarboxyaldehyde with 2-(1-methylhydrazine)pyridine was performed. At two-necked round-bottomed flask the 2-(1-methylhydrazine)pyridine (0.510g, 4.15 mmol) was weighed and placed under argon atmosphere. 4-imidazolecarboxyaldehyde (0.399 g, 4.15 mmol) was dissolved in anhydrous ethanol and was added to the reaction mixture. Yellow solution formed immediately and the reaction mixture was stirred for 24 h at 60 °C. The clear and orange solution was concentrated to minimal amount of volume, then Et_2_O was added and it was left in the refrigerator. The subsequent pale pink precipitate was filtered by vacuum filtration, washed with anhydrous ethanol and dried under vacuum to give 0.607 g (3.01 mmol) of ligand. 10 mL of Et_2_O was added to the supernatant and next part of precipitate was obtained 0.053 g (0.27 mmol). Total yield is 79.2% (0.660 g, 3.28 mmol).

ESI-MS(+) *m/z* (%): 202 (20) [H**L^4^**]^+^, 224 (25) [Na**L^4^**]^+^; ESI-MS(−): 200 (100) [**L^4^** − H]^−^. IR: ν(N-H)_br_ 3300–2600; ν(C-H)_imin._ 3011; ν(CH_3_) 2951; ν(C=C)_ar._ 1588, 1565, 1542; ν(C-N) 1475, 1440; ν(C=N)_ar_ 1309; ρ(C-H)_ar._ 1199, 1121; γ(C-H)_ar._ 891, 768, 644, 594 cm^−1^. ^1^H-NMR (CDCl_3_, 300 MHz): δ (ppm) 8.18 (d, 1H, *J* = 4.3 Hz, H_a_); 7.66 (s, 1H, H_f_); 7.61 (s, 1H, H_h_); 7.56–7.47 (m, 2H, H_c, d_); 7.23 (s, 1H, H_g_); 6.73 (t, 1H, *J* = 5.0 Hz, H_b_); 6.40 (s, broad, NH); 3.59 (s, Me_(e)_). ^13^C-NMR (CDCl_3_, 75 MHz): δ (ppm) 157.4, 147.0, 137.7, 136.0, 131.6, 125.6, 125.4, 115.6, 109.7, 29.7. Melting temperature: 163–165 °C.

- for **L^5^**: condensation of 2-imidazolecarboxyaldehyde with 2-(1-methylhydrazine)pyridine was performed. At two-necked round-bottomed flask the 2-(1-methylhydrazine)pyridine (0.525 g, 4.26 mmol) was weighed and placed under argon atmosphere. 2-Imidazolecarboxyaldehyde (0.469 g, 4.26 mmol) was dissolved in anhydrous ethanol and was added to the reaction mixture. Yellow solution formed immediately and the reaction mixture was stirred for 24 h at 60 °C. The clear and yellow solution was concentrated to minimal amount of volume and left in the refrigerator. The subsequent yellow precipitate was filtered by vacuum filtration, washed with anhydrous ethanol and dried in the vacuum to give 0.301 g (1.49 mmol). The supernatant was concentrated to minimal amount of volume and left in the refrigerator. The next part of precipitate was obtained 0.052 g (0.26 mmol). Total yield is 41.1% (0.353 g, 1.75 mmol).

ESI-MS(+) *m/z* (%): 202 (30) [H**L^5^**]^+^, 224 (100) [Na**L^5^**]^+^; ESI-MS(−): 200 (100) [**L^5^** − H]^−^. IR: ν(N-H) 3149; ν(C-H)_imin._ 3075; ν(CH_3_) 3002; ν(C=C)_ar._ 1591, 1566; ν(C-N) 1475, 1448; ν(C=N)_ar._ 1288; ρ(C-H)_ar_ 1213, 1124; γ(C-H)_ar._ 852, 773, 751, 738 cm^−1^. ^1^H-NMR (CD_3_OD, 300 MHz): δ (ppm) 8.18 (d, 1H, *J* = 5.0 Hz, H_a_); 7.83 (d, 1H, *J* = 8.6 Hz, H_d_); 7.67 (t, 1H, *J* = 7.9 Hz, H_c_); 7.62 (s, 1H, H_f_); 7.10 (s, 2H, H_g_, H_h_); 6.87 (t, 1H, *J* = 5.5 Hz, H_b_); 3.61 (s, Me_(e)_). ^13^C-NMR (CD_3_OD, 75 MHz): δ (ppm) 157.2, 149.9, 146.5, 145.1, 137.6, 124.2, 115.9, 109.9, 99.9, 28.6. Melting temperature: 191–193 °C.

- for **L^6^**: synthesized according to our previous work [31]

### 3.3. Synthesis of Complexes

#### 3.3.1. Synthetic Method for ‘Open’ Complexes

For complexes **1, 4**–**6** [**Fe**(**L^x^**)]**Cl_3_** the molar ratio of the ligand to the corresponding salt was 1:1. To a solution of **L^1^**, **L^4^**, **L^5^**, **L^6^** in MeOH the methanolic solution of FeCl_3_∙6H_2_O was added. Mixture was stirred for 24h at room temperature. Then solvents were evaporated under vacuum to minimal amount of them and Et_2_O was added. Obtained precipitate was filtrated and washed twice with Et_2_O (10 mL in total) and dried in vacuum. Yields based on ligands are shown in Table 3.

For complexes **2** and **3** reaction system was evacuated on the vacuum/gas line. To a solution of **L^2^** and **L^3^** in dry MeCN (dried on molecular sieves) FeCl_3_ ∙ 6H_2_O was added. Dark-green solution formed instantly and the reaction mixture was stirred for 48 h at room temperature. The green precipitate was filtered via suction filtration and dried in the vacuum. Yields based on ligands are shown in Table 3.

Crystals suitable for X-ray characterization were grown by vial-to-vial diffusion of different external precipitating co-solvents into the methanolic solutions of complexes at 4 °C. Please note that thoroughly dried samples were used for catalytic and biological studies, hence elementary analysis does not account for crystallization solvents that were found in the X-ray structures.

**1** [FeL^1^Cl_3_]: ESI-MS(+) *m/z* (%): 241 (100) [H**L^1^**]**^+^**; ESI-MS(−) *m/z* (%): 239 (100) [**L^1^** − H]^−^. Anal. calc. for [Fe(C_12_H_12_N_6_)(Cl)_3_] (402.48): C: 35.81; H, 3.01; N, 20.88; found: C: 35.77; H, 3.31; N, 20.30%. IR: ν(C-H)_arom_ 3125, 3093; ν_as_(C-H)_alif_ 2992, 2924; ν_s_(C-H)_alif_ 2849; ν(C=C) 1788, 1578, 1473; ν(C=N) 1437, 1350 δ(CH_3_) 1327, ν(C-O) 1289, 1245, 1214, 1148 γ(C-H)_arom_ 1085, 1048, 1008, 972, 931, 907, 850, 806, 756, 675, 615 cm^−1^.

**2** [FeL^2^Cl_2_(MeOH)]Cl*:* ESI-MS(+) *m/z* (%): 241 (70) [H**L^2^**]**^+^**, 366 (30) [Fe**L^2^**Cl_2_]^+^; ESI-MS(−) *m/z* (%): 198 (100) [FeCl_4_]^−^. Anal. calc. for [Fe(C_12_H_12_N_6_)(Cl)_2_(MeOH)]Cl (434.52): C: 35.93, H: 3.71, N: 19.34%; found: C: 35.70, H: 3.45, N: 19.77%. IR: v(N-H) 3419, ν(C-H)_arom_ 3145, 3057; ν_as_(C-H)_alif_ 2973; ν_s_(C-H)_alif_ 2938; ν(C=C) 1611, 1577, 1552, 1476, 1462; ν(C=N) 1438 δ(CH3) 1421, 1364 ν(C-O) 1314, 1287, 1255, 1216, 1180 γ(C-H)_arom_ 1119, 1083, 1054, 1008, 971, 883, 810, 753, 710, 651, 603 cm^−1^.

**3** [FeL^3^Cl_3_]: ESI-MS(+) *m/z* (%): 255 (40) [H**L^3^**]^+^, 277 (5) [Na**L^3^**]^+^, 344 (30) [Fe(**L^3^** − H)Cl]^+^. Anal. calc. for [Fe(C_13_H_14_N_6_)(Cl)_3_] (416.49): C: 37.49, H: 3.39, N: 20.18%; found: C: 37.75, H: 3.39, N: 20.41%. IR: ν(N-H) 3546,ν(C-H)_ar_ 3011; ν (C=C) 1575, 1495, 1476, 1463; ν(C=N) 1315 ρ(C-H) 1258, 1057, 1043, 1004, 979; γ(C-H) 869, 756 cm^−1^.

**4** [FeL^4^Cl_3_]: ESI-MS(+) *m/z* (%): 202 (100) [H**L^4^**]**^+^**; ESI-MS(−) *m/z* (%): 200 (100) [**L^4^** − H]^−^. Anal. calc. for [Fe(C_10_H_11_N_5_)(Cl)_3_] (363.44): C, 33.05; H, 3.05; N, 19.27; found: C, 32.96; H, 3.09; N, 19.90%. IR: ν(C-H)_arom_ 3125, 3096, 3026; ν_as_(C-H)_alif_ 2913; ν_s_(C-H)_alif_ 2862; ν(C=C) 1596, 1560, 1470; ν(C=N) 1430 δ(CH_3_) 1321, ν(C-O) 1261, 1217, 1174 γ(C-H)_arom_ 1084, 995, 958, 918, 906, 846, 774, 648, 610 cm^−1^.

**5** [FeL^5^Cl_3_]·CH_3_CN*:* ESI-MS(+) *m/z* (%): 202 (40) [H**L^5^**]**^+^**, 327 (20) [Fe**L^5^**Cl_2_]^+^; ESI-MS(−) *m/z* (%): 198 (40) [FeCl_4_]^−^. Anal. calc. for [Fe(C_10_H_11_N_5_)(Cl)_3_]·CH_3_CN (404.49): C, 35.63; H, 3.49; N, 20.78; found: C, 35.55; H, 3.40; N, 20.65%. IR: v(N-H) 3505 ν(C-H)_arom_ 3154, 3128, 3078, 3044; ν_as_(C-H)_alif_ 2917; ν_s_(C-H)_alif_ 2892; ν(C=C) 1608, 1574, 1481; ν(C=N) 1420, 1388 δ(CH_3_) 1310, ν(C-O) 1255, 1224, 1165 γ(C-H)_arom_ 1083, 1015, 869, 848, 767, 710, 626 cm^−1^.

**6** [FeL^6^Cl_3_]: ESI-MS(+) *m/z* (%): 216 (100) [H**L^6^**]**^+^**. Anal. calc. for [Fe(C_11_H_13_N_5_)(Cl)_3_] (377.46): C, 35.00; H, 3.47; N, 18.55; found: C, 33.96; H, 3.51; N, 18.30%. IR: v(N-H) 3552 ν(C-H)_arom_ 3149, 3097; ν_as_(C-H)_alif_ 2917; ν_s_(C-H)_alif_ 2846; ν(C=C) 1636, 1593, 1520, 1488; ν(C=N) 1442 δ(CH_3_) 1325 ν(C-O) 1300, 1219, 1166 γ(C-H)_arom_ 1116, 1016, 871, 778, 710, 621 cm^−1^.

#### 3.3.2. Synthesis Method for ‘Closed’ Complexes

For complexes **7**–**12** [Fe(L^x^)_2_](OTf)_2_ the molar ratio of the ligand to the corresponding salt was 2:1. To a solution of **L^1^**–**L^6^** in MeOH (MeCN/MeOH; 1:1, v:v; in case of ligand **L^1^**) the methanolic solution of Fe(OTf)_2_ was added. Mixture was stirred for 48h at room temperature. Then solvents were evaporated under vacuum to the minimal amount and Et_2_O was added. Obtained precipitate was filtrated via suction filtration and washed twice with Et_2_O (10 mL in total) and dried under vacuum. Yields based on ligands are shown in Table 3.

Crystals suitable for X-ray characterization were grown by vial-to-vial diffusion of different external precipitating co-solvents into the methanolic **7**–**10, 12** solution of complexes at 4 °C.

**7***[Fe(L^1^)_2_](CF_3_SO_3_)_2_·C_4_H_10_O:* ESI-MS(+) *m/z* (%): 241 (40) [H**L^1^**]**^+^**, 263 (5) [Na**L^1^**]^+^, 268 (80) [Fe**L^1^**_2_]^2+^, 535 (35) [Fe**L^1^**(**L^1^** − H)]^+^; ESI-MS(−): 149 (100) [CF_3_SO_3_]^−^. Anal. calc. for [Fe(C_12_H_12_N_6_)_2_](CF_3_SO_3_)_2_·C_4_H_10_O (908.66): C: 39.66, H: 3.77, N: 18.50%; found: C: 39.59, H: 3.84, N: 19.05%. IR: ν(C-H)_ar_ 3200, 3056; ν(C=C) 1640, 1575, 1480, 1475; ν(C=N) 1354, 1340; ν_as_(CF_3_SO_3_) 1240, 1225; ν_s_(CF_3_SO_3_) 1180; ρ(C-H) 1134, 1104; γ(C-H) 750, 640 cm^−1^.

**8***[Fe(L^2^)_2_](CF_3_SO_3_)_2:_* ESI-MS(+) *m/z* (%): 241 (75) [H**L^2^**]**^+^,** 263 (5) [Na**L^2^**]^+^, 268 (80) [Fe**L^2^**_2_]^2+^, 535 (95) [Fe**L^2^**(**L^2^** − H)]^+^. Anal. calc. for [Fe(C_12_H_12_N_6_)_2_](CF_3_SO_3_)_2_ (834.54): C: 37.42, H: 2.90, N: 20.14%; found: C: 37.60, H: 2.98, N: 20.70%. IR: ν(N-H) 3492; ν(C-H)_ar_ 3154, 3100, 2948; ν(C=C) 1640, 1575, 1480, 1475; ν(C=N) 1390; ν_as_(CF_3_SO_3_) 1248, 1225; ν_s_(CF_3_SO_3_) 1160; ρ(C-H) 1118, 1052, 1030, 1008; γ(C-H) 750, 640 cm^−1^.

**9***[Fe(L^3^)_2_](CF_3_SO_3_)_2_·C_5_H_12_O:* ESI-MS(+) *m/z* (%): 282 (95) [Fe**L^3^**_2_]^2+^, 563 (70) [Fe**L^3^**(**L^3^** − H)]^+^; ESI-MS(−): 149 (100) [CF_3_SO_3_]^−^. Anal. calc. for [Fe(C_13_H_14_N_6_)_2_](CF_3_SO_3_)_2_·C_5_H_12_O (950.74): C: 41.69, H: 4.24, N: 17.68%; found: C: 41.52, H: 4.41, N: 17.25%. IR: ν(N-H) 3475; ν(C-H)_ar_ 3120, 2954; ν(C=C) 1640, 1560, 1480, 1475; ν(C=N) 1370; ν_as_(CF_3_SO_3_) 1250, 1238; ν_s_(CF_3_SO_3_) 1152; ρ(C-H) 1054, 1030; γ(C-H) 750, 642 cm^−1^.

**10***[Fe(L^4^)_2_](CF_3_SO_3_)_2_·C_4_H_10_O:* ESI-MS(+) *m/z* (%): 229 (100) [Fe**L^4^**_2_]^+^, 457 (35) [Fe**L^4^**(**L^4^** − H)]^+^; ESI-MS(−): 149 (100) [CF_3_SO_3_]^−^. Anal. calc. for [Fe(C_10_H_11_N_5_)_2_](CF_3_SO_3_)_2_·C_4_H_10_O (830.56): C: 37.60, H: 3.88, N: 16.86 %; found: C: 37.71, H: 3.57, N: 16.54%. IR: ν(N-H) 3208, 3185; ν(C-H)_ar_ 3118, 2910; ν(C=C) 1608, 1575, 1550, 1500; ν(C=N) 1315; ν_as_(CF_3_SO_3_) 1248, 1230; ν_s_(CF_3_SO_3_) 1151; ρ(C-H) 1090, 1032; γ(C-H) 765, 647 cm^−1^. ^1^H-NMR (CD_3_CN, 300 MHz): δ (ppm) 10.85 (s, 2H); 9.12 (s, 2H); 7.64–7.40 (m, 6H); 7.10 (s, 2H); 7.01 (d, 2H); 6.71 (t, 2H); 4.18 (s, 6H).

**11***[Fe(L^5^)_2_](CF_3_SO_3_)_2:_* ESI-MS(+) *m/z* (%): 229 (100) [Fe**L^5^**_2_]^2+^, 457 (20) [Fe**L^5^**(**L^5^** − H)]^+^; ESI-MS(−): 149 (100) [CF_3_SO_3_]^−^. Anal. calc. for [Fe(C_10_H_10_N_5_)_2_](CF_3_SO_3_)_2_ (756.44): C: 34.93, H: 2.93, N: 18.52 %; found: C: 34.10, H: 3.09, N: 18.02%. IR: ν(N-H) 3540, 3480; ν(C-H)_ar_ 3175, 3110, 3055; ν (C=C) 1610, 1576, 1540, 1500, 1418; ν(C=N) 1352; ν_as_(CF_3_SO_3_^−^) 1248; ν_s_(CF_3_SO_3_) 1151; ρ(C-H) 1030; γ(C-H) 850, 759 cm^−1^. ^1^H-NMR (CD_3_CN, 300 MHz): δ (ppm) 9.28 (s, 2H); 7.69–7.52 (m, 4H); 7.06 (d, 2H); 7.01 (s, 2H); 6.77 (t, 2H); 6.37 (s, 2H); 4.28 (s, 6H).

**12***[Fe(L^6^)_2_](CF_3_SO_3_)_2_·CH_3_OH:* ESI-MS(+) *m/z* (%): 243 (100) [Fe**L^6^**_2_]^2+^, 635 (30) [Fe**L^6^**_2_(CF_3_SO_3_)]^+^; ESI-MS(−): 149 (100) [CF_3_SO_3_]^−^. Anal. calc. for [Fe(C_11_H_13_N_5_)_2_](CF_3_SO_3_)_2_·CH_3_OH (816.56): C: 36.77, H: 3.70, N: 17.15%; found: C: 36.66, H: 3.38, N: 17.50%. IR: ν(C-H)_ar_ 3120, 2958; ν(C=C) 1612, 1570, 1546, 1500, 1448; ν(C=N) 1338; ν_as_(CF_3_SO_3_) 1254, 1237; ν_s_(CF_3_SO_3_) 1151; ρ(C-H) 1125, 1035; γ(C-H) 760, 648 cm^−1^. ^1^H-NMR (CD_3_CN, 300 MHz): δ (ppm) 9.26 (s, 2H); 7.69 (t, 2H); 7.56 (d, 2H); 7.03 (d, 2H); 6.89 (s, 2H); 6.75 (t, 2H); 6.29 (s, 2H); 4.28 (s, 6H); 3.92 (s, 6H).

### 3.4. X-ray Crystallography

Diffraction data were collected by the ω-scan technique at 100(1)K (**2**, **6**–**10**,**12**) on Rigaku XCalibur four-circle diffractometer with EOS CCD detector and graphite-monochromated MoKα radiation (λ = 0.71073 Å) and at 130(1)K (**1**, **4**) on Rigaku SuperNova four-circle diffractometer with Atlas CCD detector and mirror-monochromated CuKα radiation (λ = 1.54178 Å). The data were corrected for Lorentz-polarization as well as for absorption effects [59]. Precise unit-cell parameters were determined by a least-squares fit of the reflections of the highest intensity, chosen from the whole experiment. The structures were solved with SHELXT-2013 [60] and refined with the full-matrix least-squares procedure on F2 by SHELXL-2013 [61]. All non-hydrogen atoms were refined anisotropically, hydrogen atoms were placed in idealized positions and refined as ‘riding model’ with isotropic displacement parameters set at 1.2 (1.5 for CH_3_) times Ueq of appropriate carrier atoms. The crystals of **1** were of very poor quality, and the diffraction was measurable only for low angles. Therefore the number of data is low and the C and N atoms were refined isotropically. However, as this was the only example of the complex of its class, and the geometry looks quite reasonable, we decided to include this structure in the manuscript. Structures of five other complexes (generally, with large voids with unidentified electron density) were deposited in the CDB and attached as Supplementary material. Alerts B in **7**, **8**, **9**, **10** and **12** are related to the lack of certain (small) number of reflections, caused by the experimental conditions, or (for **12**) by high value of Flack parameter. In this case, we have tried the twin/basf refinement but the results are slightly inferior (even though the Alert vanishes). Crystallographic data for the structural analysis has been deposited with the Cambridge Crystallographic Data Centre, deposition numbers are included in Table 4, which is shown below. Copies of this information may be obtained free of charge from: The Director, CCDC, 12 Union Road, Cambridge, CB2 1EZ, UK; e-mail: deposit@ccdc.cam.ac.uk, or www: www.ccdc.cam.ac.uk.

### 3.5. Catalytic Oxidation of 2-Aminophenol

Catalytic oxidation of o-aminophenol Phenoxazinone synthase like activity of our synthesized ‘open’ and ‘close’ system complexes was investigated by the reaction of 2.0 × 10^−5^ M methanolic solutions of the complexes with 2.0 × 10^−4^ M methanolic solution of o-aminophenol (OAPH) at room temperature and in the presence of air. The reactions were monitored on spectrophotometer by increasing absorbance at ca. 433 nm which correspond to band from the product of reaction, phenoxazinone chromophore (2-aminophenoxazine-3-one). Each measurement was performed three times and given values represent a representative average.

Determination of different kinetic parameters for the catalytic activity was done using the procedure reported earlier [25,41] based on Michaelis– Menten model. It gave a double reciprocal Lineweaver–Burk plots on which values of V_max_ were interpreted, values of K_M_ were from ½ V_max_ on *x*-axis, while values of k_cat_ is results of V_max_/[catalyst].

### 3.6. DNA Binding Assays

#### 3.6.1. Absorption Titration

The absorbance titrations were performed in fixed concentration of metal complexes (20 µM) while gradually increasing the concentration of CT-DNA within the range from 0 to 100 µM. Each sample solution was allowed to equilibrate 5 min. before the spectra were recorded. Using the absorption titration data, the binding constant K_b_ was determined according to the equation [62]:

[DNA]/(ε_a_ − ε_f_) = [DNA]/(ε_b_ − ε_f_) +1/K_b_ (ε_b_ − ε_f_)

where [DNA] is the concentration of CT-DNA in the base pairs, ε_a_ corresponds to the extinction coefficient observed (A_obsd_/[M]), ε_f_ corresponds to coefficient of free compound, ε_b_ is the extinction coefficient of the compound fully bound to CT-DNA, and K_b_ is the intrinsic binding constant. The K_b_ value was given by the ratio of slope to intercept in the plot of [DNA]/(ε_a_ − ε_f_) versus [DNA].

The standard Gibb’s free energy for DNA binding was calculated based on the equation [63]:

$$G_{h}^{0}=\;-{\mathrm{RTlnK}}_{b}$$

where $\Delta G_{h}^{0}$ is the standard Gibb’s free energy, R corresponds to gas constant, T is temperature and K_b_ stands for binding constant of the appropriate complex.

#### 3.6.2. Competitive Binding Fluorescence Experiment

The competitive binding of compounds with ethidium bromide (EB) has been investigated by the fluorescence emission spectroscopy in order to evaluate its ability to displace EB from its DNA–EB fluorescent complex. The solution of EB (20 µM) and CT-DNA (26 µM) was allowed to equilibrate for 30 min. at 25 °C before the measurements were taken. While increasing the concentration of investigated complexes, the fluorescence spectra were recorded in the range of 540–720 nm at λ_exc_ = 467 nm. The ability of compounds to quench the emission of DNA-EB complex was evaluated by the Stern–Volmer equation [64]:

I_0_/I = 1 + K_SV_

were I_0_ and I are fluorescence intensities in absence and presence of compound, respectively, and K_SV_ is the Stern–Volmer constant. K_SV_ depends on the ratio of bound EB to the concentration of CT-DNA.

#### 3.6.3. Circular Dichroism Studies

For the CD experiments 2 µM oligo DNA d(GTTAATCGCTGG) as a solution in Tris Buffer (10 mM TrisHCl, 5 mM NaCl, 50 mM pH 7.5) was used. 1–30 eq. of complexes were added to the solution 5 min. before measurements. At 1:30 molar ratio the samples were annealed at 70 °C for 5 min. and then slowly cooled down to room temperature. Quartz cuvettes with a path length of 1 cm were used with the sample volumes of 2 mL. Spectra were collected in the range 320–220 nm at 20 °C and were averaged over three scans.

## 4. Conclusions

Synthesis of a small library of Schiff base ligands is presented, which were designed so as to behave as efficient tridentate chelators with simultaneous incorporation of different number and disposition of non-coordinating NH moieties. Information encoded within iron(II/III) metallic centres and counterions allowed for generation of two families of complexes, which we term as ‘open’: [Fe(L^x^)Cl_3_] and ‘closed’: [[Fe(L^x^)_2_](OTf)_2_. Complexes were studied as *phenoxazinone synthase* activity mimics and DNA-binders, with the aim to see if there is an effect of hydrogen bonding on the studied biological activity.

Catalytic studies and derived kinetic parameters of 2-aminophenol (OAPH) oxidation with synthesized complexes show that the number and disposition of NH moieties influences the initial binding of OAPH to the complex. Nonetheless, its subsequent oxidation to 2-aminophenoxazine-3-one chromophore (APX) and thus catalytic activity is an outcome of additional factors. Consequently, attributing their activity solely to H-bonding is inaccurate. We postulate however that the mechanism of action differs within the ‘open’ and ‘closed’ families, and effect of non-covalent interactions is more important in the latter case.

The more straightforward effect of employing benzimidazole and imidazole moieties in the ligands scaffold was observed for DNA-binding studies. Intriguingly, the family of ‘open’ [Fe(L^x^)Cl_3_] complexes do not interact with studied nucleic acid, which can be attributed to the effect of negatively charged chlorides, which may repel the complex from the sugar-phosphate backbone of CT-DNA. In contrast, ‘closed’ [Fe(L^x^)_2_](OTf)_2_ complexes exhibit the behavior of bulky cations that are able to interact with the negatively charged backbone of DNA. Electronic absorption titration, fluorescence competitive binding with EB and CD titration allowed one to confirm that from the group of triflate analogues **7**–**12**, only the pyridine-scaffolded ‘closed’ species **11** and **12** effectively bind with DNA, most probably by the intercalation-type mode. This shows that the benzimidazole moiety effectively precludes the remaining species from intercalation or any other significant interaction with nucleic acids and the imidazole substituent can be used for tuning of the interaction strength. In our previous works [38,40,65], a similar phenomenon was observed, where complexes without hydrogen bonding donors in the “primary” structure bind to DNA scaffold—such as helical and “open” complexes with terpyridine-type and quaterpyridine-type ligands [66].

We are currently working on ways to introduce H-bonding moieties in the immediate vicinity of the compounds’ coordination sphere as well as for utilization of the above-demonstrated phenomenon on aspects of crystal engineering and spin-crossover magnetic properties.

## Figures and Tables

**Table 1:** Kinetic parameters for complexes **1–12**.

Catalyst	V_max_ (10^−3^Ms^−1^)	K_M_ (10^−3^M)	k_cat_ (h^−1^)
[Fe(**L^1^**)Cl_3_] (**1**)	3.71	1.45	185.25
[Fe(**L^2^**)Cl_3_] (**2**)	2.55	2.16	127.30
[Fe(**L^3^**)Cl_3_] (**3**)	3.45	1.95	172.30
[Fe(**L^4^**)Cl_3_] (**4**)	2.68	2.02	134.05
[Fe(**L^5^**)Cl_3_] (**5**)	2.07	2.02	103.34
[Fe(**L^6^**)Cl_3_] (**6**)	3.01	2.19	150.49
[Fe(**L^1^**)_2_](OTf)_2_ (**7**)	3.98	1.56	199.40
[Fe(**L^2^**)_2_](OTf)_2_ (**8**)	1.96	2.08	97.68
[Fe(**L^3^**)_2_](OTf)_2_ (**9**)	3.68	1.86	183.80
[Fe(**L^4^**)_2_](OTf)_2_ (**10**)	0.86	1.79	42.76
[Fe(**L^5^**)_2_](OTf)_2_ (**11**)	1.30	1.89	65.14
[Fe(**L^6^**)_2_](OTf)_2_ (**12**)	2.13	2.08	106.53

**Table 2:** Catalytic oxidation of o-aminophenol with transition metal complexes.

Catalyst	Solvent	k_cat_ (h^−1^)	Ref.
[Fe(**L^1^**)Cl_3_] (**1**)	Methanol	185.25	This work
[Fe(**L^1^**)_2_](OTf)_2_ (**7**)	Methanol	199.40	This work
[FeCl_2_(**L^a^**)]	DMF	137.0	[28]
[Fe(**L^b^**)Cl_3_]	Methanol	56.0	[20]
[{Fe(**L^c^**)(4,4′-byp)ClO_4_}]n	Methanol	32.36	[42]
[Fe**L^c^**Cl]_2_	Methanol	196.18	[42]
[Co(**L^d^**)(N_3_)_3_]	Methanol	33.26	[23]
[Co(**L^e^**)(N_3_)_2_]	Methanol	54.0	[45]
[Co(**L^f^**)Cl(H_2_O)]Cl·H_2_O	Methanol	13.68	[46]
[**L^g^**Co(**L^h^**)_2_]ClO_4_	Methanol	11.48	[47]

L^a^ = 1,3-bis(5′-methyl-2′-thiazolylimino)isoindoline; L^b^ = *N,N’*-Bis(2-Methylbenzimidazolyl) pyridinediamide; L^c^ = *N,N’*-Disalicylidene-1,2-propylenediamine; L^d^ = (2-pyridylmethyl)(2-pyridylethyl)amine; L^e^ = 2-{[3-(3-Dimethylaminopropylamino)-propylimino]-methyl}-6-methoxy-phenol; L^f^ = *N,N*’-bis(pyridin-2-ylmethylene)-2,2-dimethylpropane-1,3-diamine; L^g^ = *N,N’*-bis(3-methoxysalicylidehydene)cyclohexane-1,2-diamine; L^h^ = 4-aminopyridine.

**Table 3:** The amount of substrate used to prepare complexes and information about yield and crystals of them.

Complex	Ligand (mg)	Fe(II/III) Salt (mg)	Precipitation Color/Solid	Yield ^a^		Crystals From
				mg	%	
**1**	26.7	30.0	very dark green	50.4	88.9	iPr_2_O
**2**	402.9	453.6	dark green	693.7	81	tBuOMe
**3**	60.0	63.8	dark green	66.2	67.3	-
**4**	22.3	30.0	dark green	46.8	89.4	iPr_2_O
**5**	23.9	30.0	black	47.3	87.7	-
**6**	21.9	30.0	dark green	39.8	76.8	iPr_2_O
**7**	70.0	51.6	cinnamon	87.9	72.3	Et_2_O
**8**	70.0	51.6	hot chocolate	104.0	72.3	iPr_2_O
**9**	70.0	48.7	raspberry	89.0	75.0	tBuOMe
**10**	70.0	61.6	red	121.0	91.9	Et_2_O
**11**	70.0	61.6	warm brown	117.0	88.8	-
**12**	55.0	45.3	dark chocolate	90.0	89.2	PhMe

^a^ yield based on powder.

**Table 4:** Crystal data, data collection and structure refinement.

Compound	1	2	4	6	7	8	9	10	12
Formula	C_12_H_12_Cl_3_FeN_6_	C_13_H_16_Cl_2_FeN_6_O^+^·Cl^−^	C_10_H_11_Cl_3_FeN_5_	C_11_H_13_Cl_3_FeN_5_	C_24_H_24_FeN_12_^2+^·2(CF_3_SO_3_)^−^·C_4_H_10_O	C_24_H_24_FeN_12_^2+^·2(CF_3_SO_3_)^−^·solvent	C_26_H_28_FeN_12_^2+^·2(CF_3_SO_3_) ^−^·C_5_H_12_O	C_20_H_22_FeN_10_^2+^·2(CF_3_SO_3_)^−^·C_4_H_10_O	C_22_H_26_FeN_10_^2+^·2(CF_3_SO_3_)^−^·CH_4_O
	Fe**L**^1^Cl_3_	[Fe**L**^2^Cl_2_(CH_3_OH)]^+^·Cl^−^	Fe**L**^4^Cl_3_	Fe**L**^6^Cl_3_	(Fe**L**^1^_2_)^2+^·2(CF_3_SO_3_)^−^·C_4_H_10_O	(Fe**L**^2^_2_)^2+^·2(CF_3_SO_3_)^−^·solvent	(Fe**L**^3^_2_)^2+^·2(CF_3_SO_3_)^−^·C_5_H_12_O	(Fe**L**^4^_2_)^2+^·2(CF_3_SO_3_)^−^·C_4_H_10_O	(Fe**L**^6^_2_)^2+^·2(CF_3_SO_3_)^−^·CH_3_OH
Formula weight	402.48	434.52	363.44	377.46	908.66	834.54	950.74	830.58	816.56
Crystal system	triclinic	monoclinic	triclinic	monoclinic	monoclinic	monoclinic	triclinic	monoclinic	orthorhombic
Space group	P-1	P2_1_/n	P-1	P2_1_/c	C2/c	P2_1_/c	P-1	P2_1_/c	Pca2_1_
a (Å)	7.459(2)	7.6840(4)	7.5198(4)	7.2144(2)	23.1912(8)	12.9879(7)	12.4297(8)	15.5382(2)	22.3563(6)
b (Å)	9.055(2)	13.9429(6)	8.1040(6)	12.4684(3)	17.0021(5)	13.0532(8)	13.0407(7)	12.55315(14)	8.3830(2)
c (Å)	12.460(3)	16.8684(6)	12.4702(10)	16.7244(4)	22.3107(8)	21.9024(19)	13.4814 (7)	18.1225 (2)	17.5420(6)
α (°)	75.69(2)	90	75.205(6)	90	90	90	88.870(4)	90	90
β (°)	87.94(2)	98.281(4)	84.655(5)	99.730(2)	116.163(4)	91.711(6)	74.131(5)	91.2230(13)	90
γ (°)	66.12(3)	90	66.519(6)	90	90	90	75.262(5)	90	90
V(Å^3^)	743.7(4)	1788.39(14)	673.87(9)	1482.75(7)	7895.8(5)	3711.5(4)	2029.9(2)	3534.05(7)	3287.60(16)
Z	2	4	2	4	8	4	2	4	4
D_x_(g cm^−3^)	1.797	1.614	1.791	1.691	1.529	1.493	1.556	1.561	1.650
F(000)	406	884	366	764	3728	1696	980	1704	1672
μ (mm^−1^)	13.136	1.305	14.392	1.553	0.577	0.604	0.565	0.634	0.680
Reflections:									
collected	1450	7518	4550	6161	18337	13346	30279	15200	17885
unique (R_int_)	917 (0.042)	3576 (0.024)	2666 (0.054)	3106 (0.022)	7987 (0.031)	6526 (0.059)	7129 (0.083)	7095 (0.015)	5695 (0.029)
with I>2σ(I)	716	2958	2319	2688	6243	3623	5726	6301	5323
R(F) [I>2σ(I)]	0.078	0.033	0.075	0.029	0.058	0.081	0.059	0.030	0.063
wR(F^2^) [I>2σ(I)]	0.233	0.081	0.196	0.059	0.174	0.153	0.156	0.072	0.165
R(F) [all data]	0.104	0.044	0.084	0.037	0.077	0.146	0.070	0.035	0.067
wR(F^2^) [all data]	0.233	0.087	0.204	0.063	0.188	0.174	0.169	0.075	0.169
Goodness of fit	1.05	1.05	1.14	1.04	1.24	1.02	1.07	1.02	1.08
max/min Δρ (e·Å^−3^)	0.77/−0.98	0.50/−0.29	1.75/−0.79	0.32/−0.31	1.68/−0.67	0.87/−0.45	1.14/−0.62	0.34/−0.39	0.83/−0.92
CCDC number	1851825	1851824	1851829	1851826	1871758	1871759	1871760	1871763	1871761

## Supplementary Materials

The following are available online: Ms PDF document with all supplementary Figures and Tables.
