# Supplementary material for: New Artificial Biomimetic Enzyme Analogues Based on Iron(II/III) Schiff Base Complexes: An Effect of (Benz)imidazole Organic Moieties on Phenoxazinone Synthase and DNA Recognition
Source: Molecules. 2019 Aug 31;24(17):3173. doi: 10.3390/molecules24173173 (PMC6749401; doi:10.3390/molecules24173173)
Supplement: Supplementary file 1 [file molecules-24-03173-s001.pdf]

Electronic Supplementary Information for

New artificial biomimetic enzyme analogues based on iron(II/III) Schiff Base complexes: an effect of (benz)imidazole organic moieties on phenoxazinone synthase and DNA recognition

Aleksandra Bocian[1#], Martyna Szymańska[1#], Daria Brykczyńska[1], Maciej Kubicki[1], Monika Wałęsa-Chorab[1], Giovanni N. Roviello[2], Marta A. Fik-Jaskółka[1*], Adam Gorczyński[1*], Violetta Patroniak[1]

[1] Faculty of Chemistry, Adam Mickiewicz University, Uniwersytetu Poznańskiego 8, 61-614 Poznań, Poland

[2] Institute of Biostructures and Bioimaging – CNR, via Mezzocannone 16, 80134 Napoli, Italy

[#]**co-authors contributed equally**

* adam.gorczynski@amu.edu.pl; martafik@amu.edu.pl

Table of contents

1. ^{1}H NMR, ^{13}C NMR spectra of ligands and complexes 2
2. ESI-MS spectra of ligand and complexes 10
3. Crystal data and structure of complexes 19
4. Spectra of the oxidation reactions 24
5. Biological spectra 28

1. ^{1}H NMR, ^{13}C NMR spectra of ligands and complexes

Fig. S1a. ^{1}H NMR spectra for ligand **L^1** measured in $(CD_3)_2SO$.

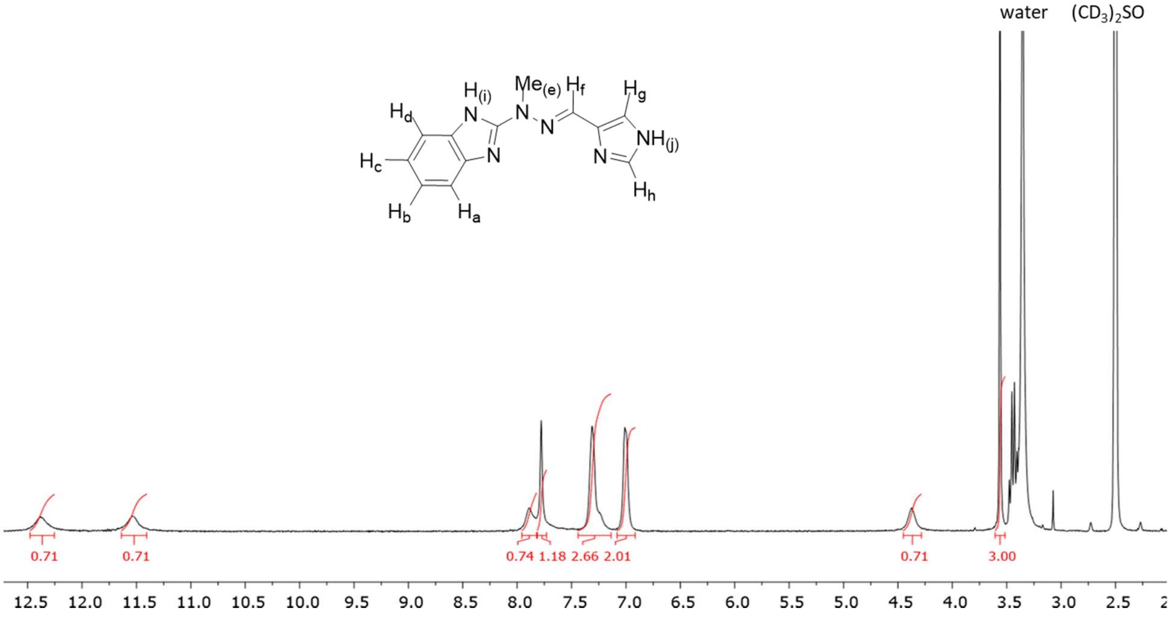

^{1}H NMR ($(CD_3)_2SO$, 300 MHz): δ (ppm) – 12.38 (s, 0.7H, $NH_{(i/j)}$); 11.53 (s, 0.7H, $NH_{(i/j)}$); 7.89 (s, 1H, H_h); 7.78 (s, 1H, H_f); 7.31 (s, 3H, $H_{b,c,g}$); 7.01 (s, 2H, $H_{a,d}$); 4.37 (s, 0.7H, $NH_{(i/j)}$); 3.56 (s, $Me_{(e)}$).

Fig. S1b. ^{1}H NMR spectra for ligand **L^1** measured in $(CD_3)_2SO$ with K_2CO_3.

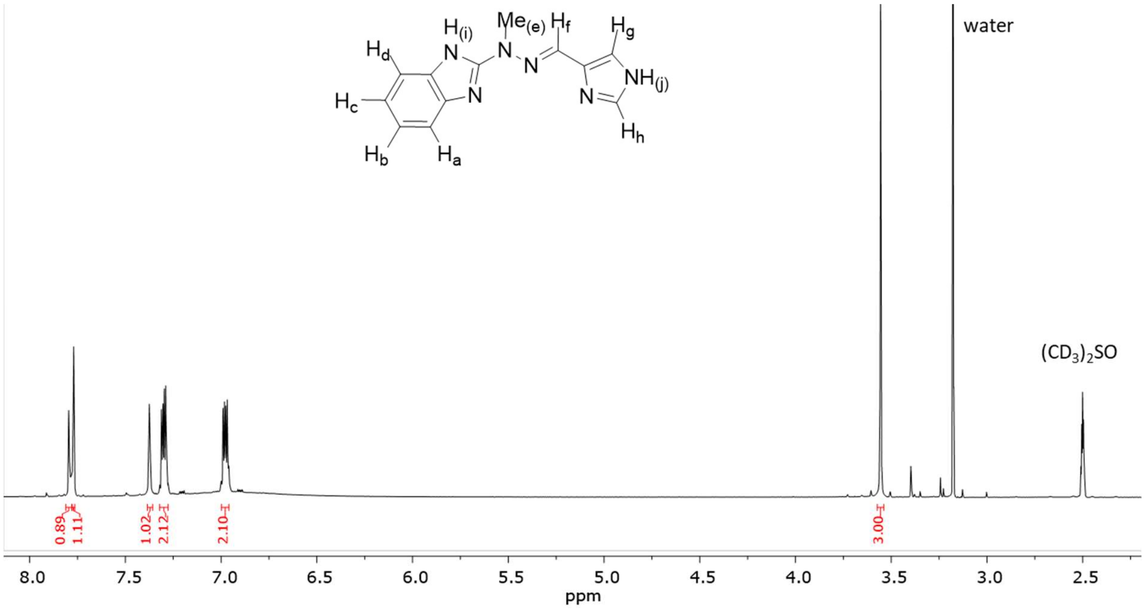

^{1}H NMR ($(CD_3)_2SO$ + K_2CO_3, 400 MHz) δ = 7.80 (s, 1H, H_h); 7.77 (s, 1H, H_f); 7.38 (s, 1H, H_g); 7.32-7.28 (dd, 2H, J = 5.8, 3.2 Hz, $H_{b,c}$); 7.00-6.96 (dd, 2H, J = 5.9, 3.2 Hz, $H_{a,d}$); 3.56 (s, $Me_{(e)}$).

Fig. S1c. Comparison of ^{1}H NMR spectra for ligand **L¹** measured in $(CD_3)_2SO$ and in $(CD_3)_2SO$ with K_2CO_3.

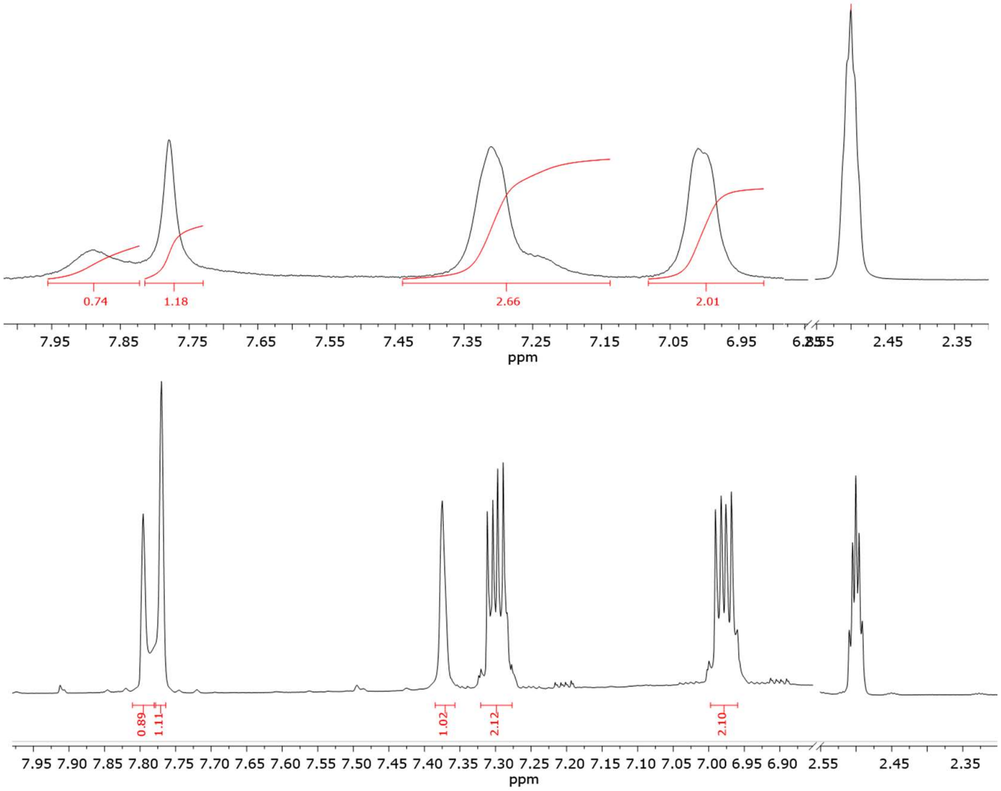

Fig. S2. ^{13}C NMR spectra for ligand **L¹** measured in $(CD_3)_2SO$.

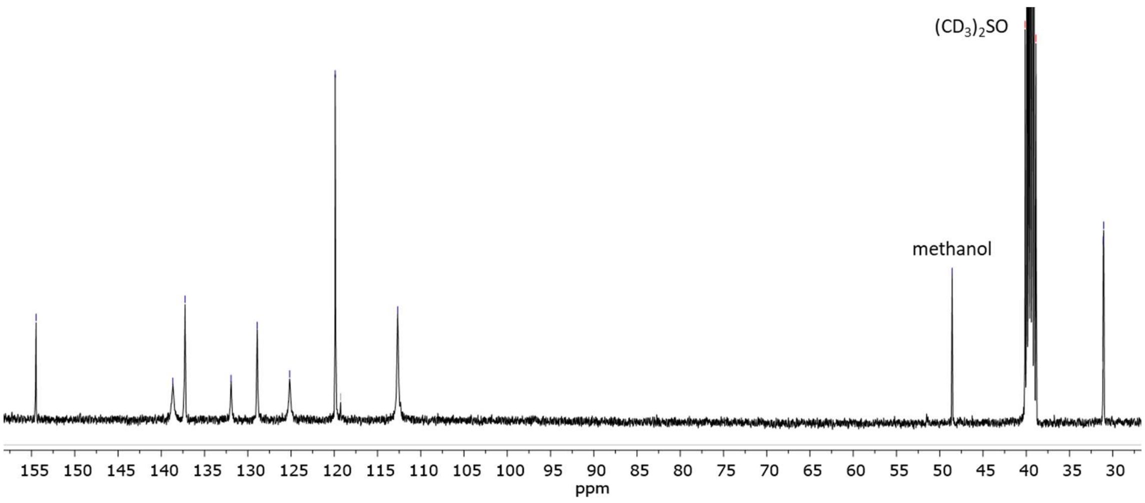

^{13}C NMR ($(CD_3)_2SO + K_2CO_3$, 75 MHz): δ (ppm) – 154.5, 138.7, 137.3, 131.9 (2C), 128.9, 125.2, 119.9 (2C), 112.7 (2C), 31.1.

Fig. S3. ^{1}H NMR spectra for ligand **L**^{2} measured in $(CD_3)_2SO$.

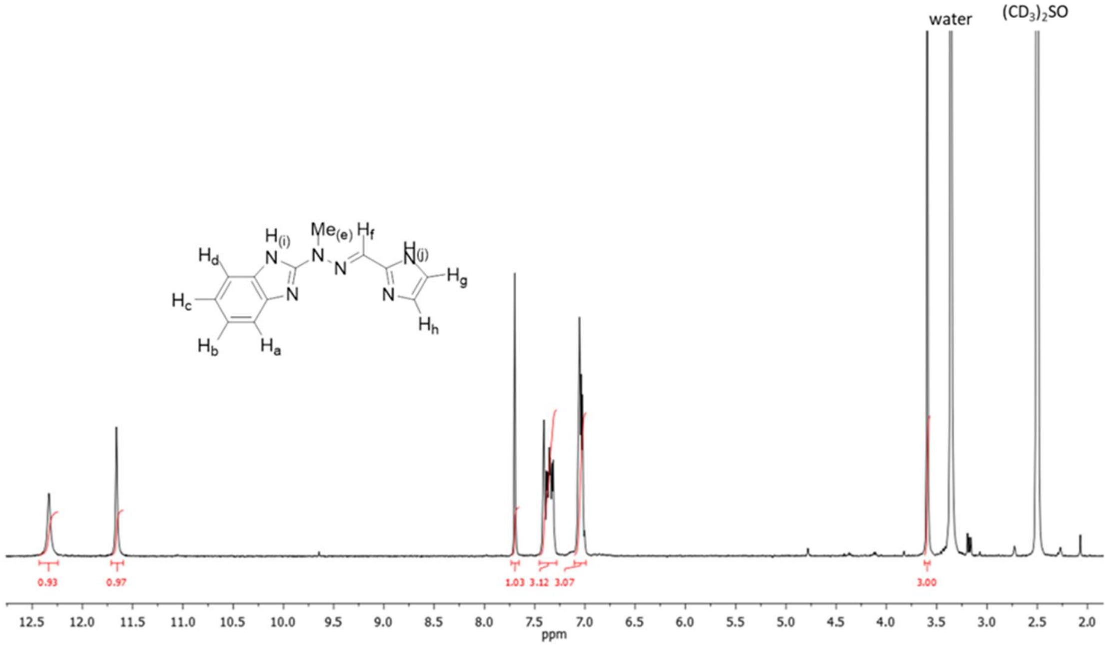

^{1}H NMR ($(CD_3)_2SO$, 300 MHz): δ (ppm) – 12.33 (s, 1H, $NH_{(i)}$); 11.66 (s, 1H, $NH_{(j)}$); 7.70 (s, 1H, H_f); 7.45-7.28 (m, 3H, $H_{b, c, g}$); 7.10-6.99 (m, 3H, $H_{a, d, h}$); 3.59 (s, $Me_{(e)}$).

Fig. S4. ^{13}C NMR spectra for ligand **L**^{2} measured in $(CD_3)_2SO$.

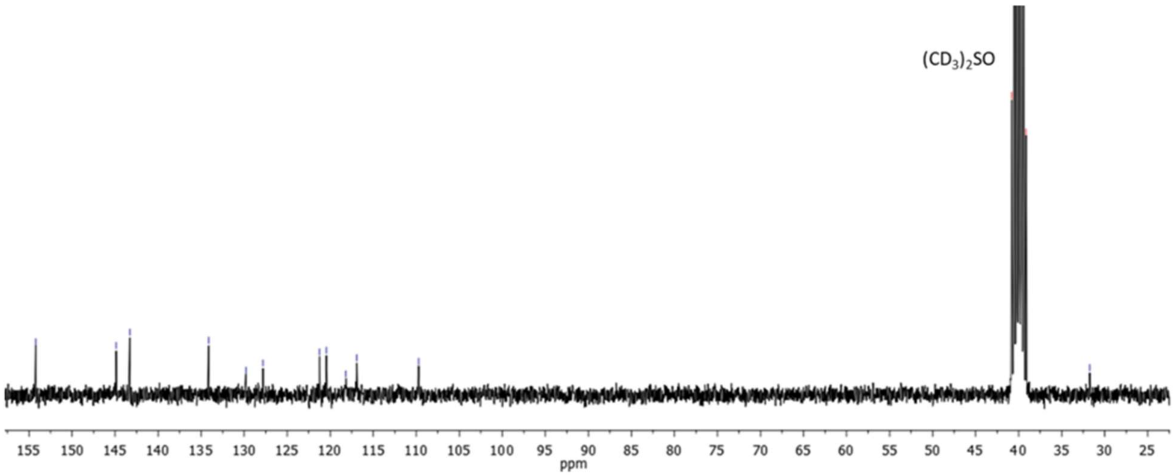

^{13}C NMR ($(CD_3)_2SO$, 75 MHz): δ (ppm) – 154.2, 144.9, 143.3, 134.1, 129.8, 127.8, 121.2, 120.4, 118.2, 116.9, 109.7, 31.7.

Fig. S5. ^{1}H NMR spectra for ligand **L**³ measured in $(CD_3)_2SO$.

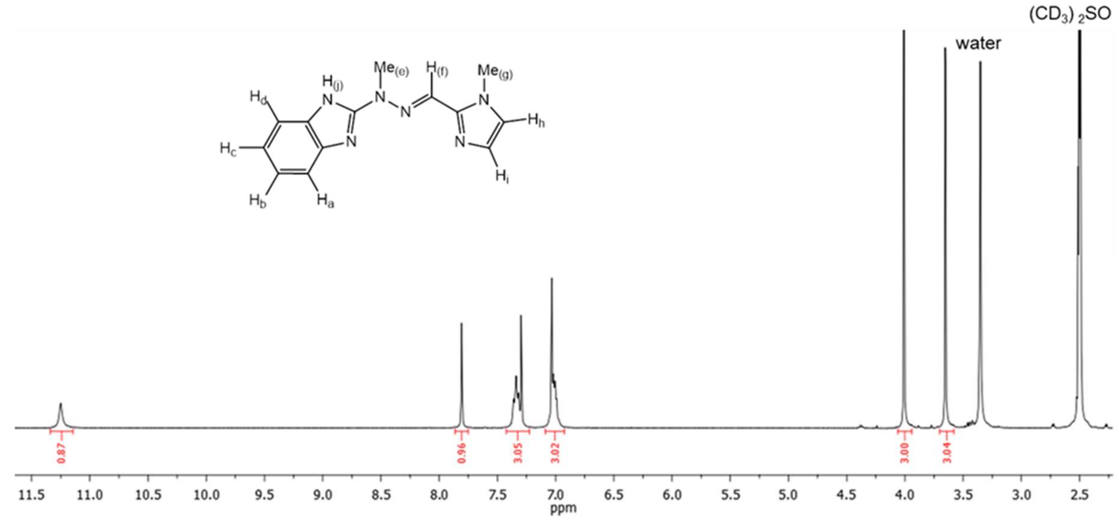

^{1}H NMR ($(CD_3)_2SO$, 300 MHz): δ (ppm) – 11.25 (s, 1H, $H_{(j)}$), 7.81 (s, 1H, $H_{(f)}$), 7.36-7.29 (m, 3H, $H_{b, c, h}$), 7.04-6.99 (m, 3H, $H_{a, d, i}$), 4.01 (s, $Me_{(g)}$), 3.65 (s, $Me_{(e)}$).

Fig. S6. ^{13}C NMR spectra for ligand **L**³ measured in $(CD_3)_2SO$.

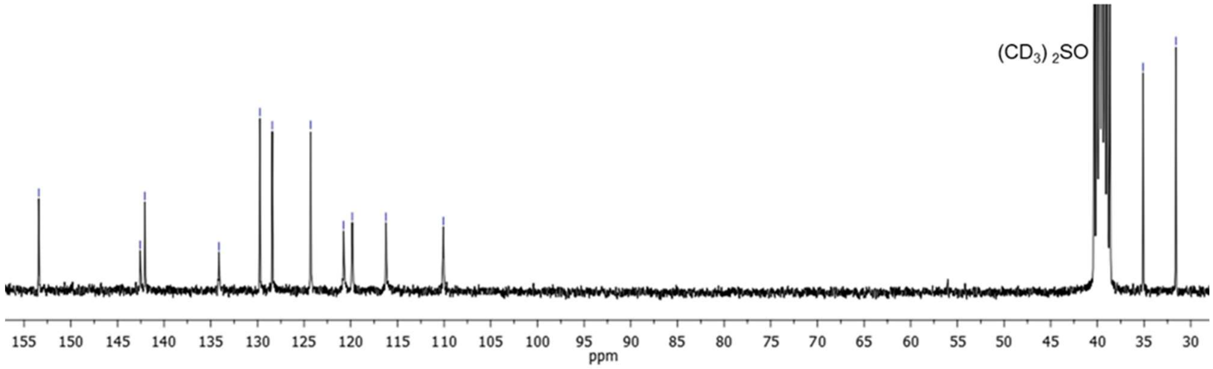

^{13}C NMR ($(CD_3)_2SO$, 75 MHz): δ (ppm) – 153.4, 142.6, 144.1, 134.1, 129.8, 128.4, 124.3, 120.8, 119.8, 116.2, 110.1, 35.1, 31.6.

Fig. S7. ^{1}H NMR spectra for ligand **L⁴** measured in $CDCl_3$.

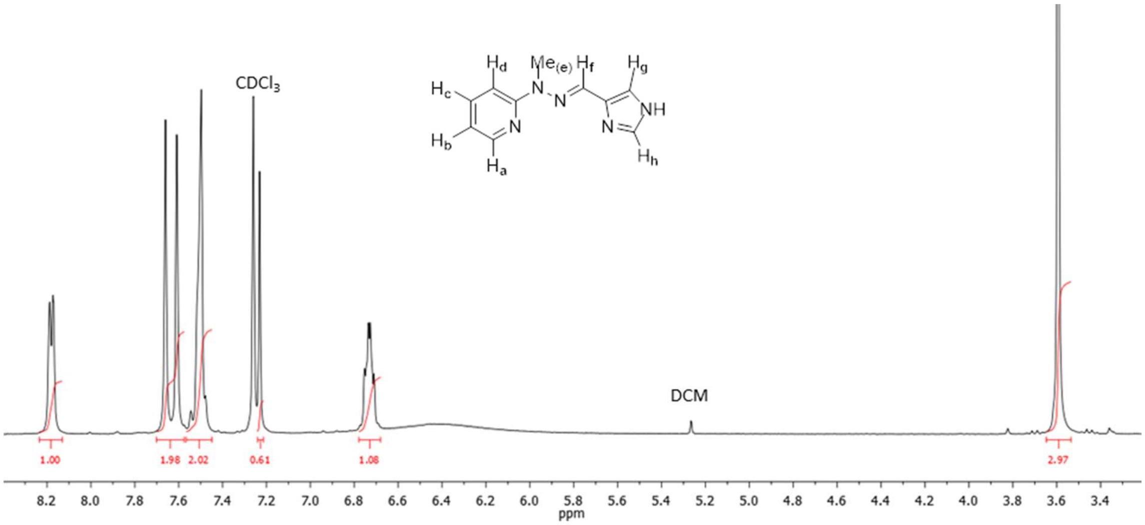

^{1}H NMR ($CDCl_3$, 300 MHz): δ (ppm) – 8.18 (d, 1H, J = 4.3 Hz, H_a); 7.66 (s, 1H, H_f); 7.61 (s, 1H, H_h); 7.56-7.47 (m, 2H, $H_{c, d}$); 7.23 (s, 1H, H_g); 6.73 (t, 1H, J = 5.0 Hz, H_b); 6.40 (s, broad, NH); 3.59 (s, $Me_{(e)}$).

Fig. S8. ^{13}C NMR spectra for ligand **L⁴** measured in $CDCl_3$.

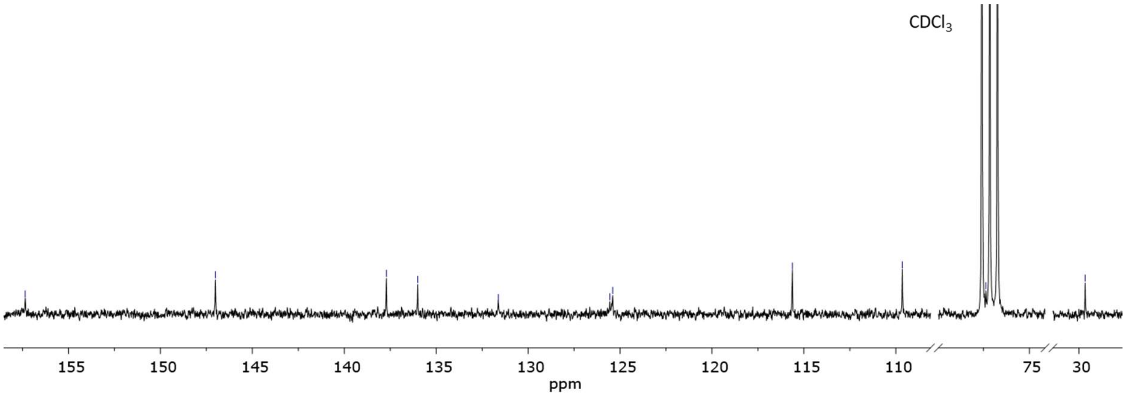

^{13}C NMR ($CDCl_3$, 75 MHz): δ (ppm) – 157.4, 147.0, 137.7, 136.0, 131.6, 125.6, 125.4, 115.6, 109.7, 29.7.

Fig. S9. ^{1}H NMR spectra for ligand **L**[5] measured in CD_3OD.

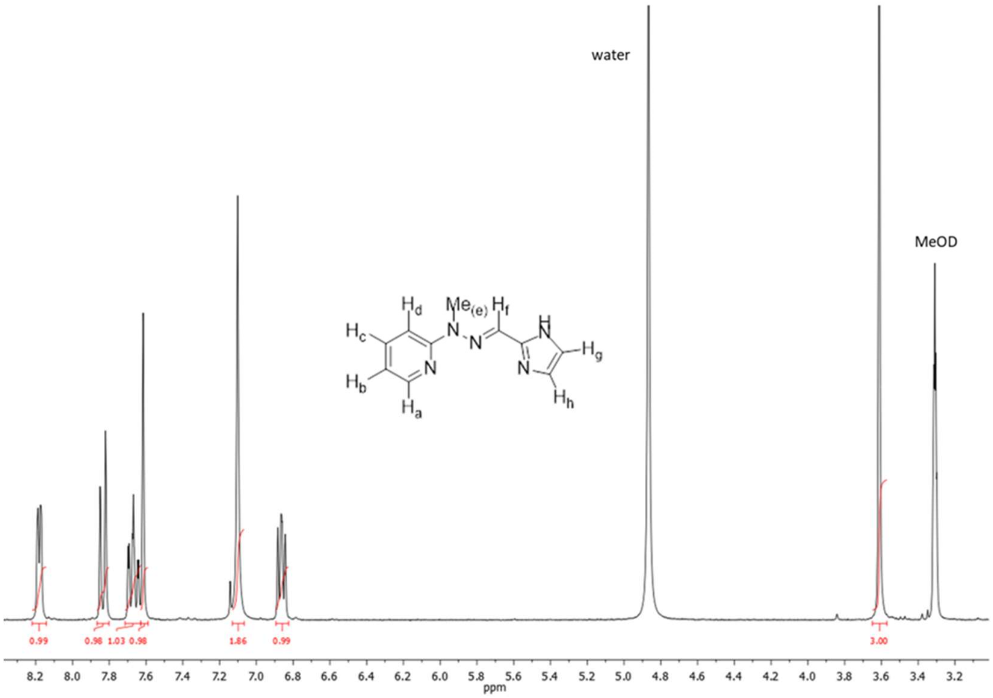

^{1}H NMR (CD_3OD, 300 MHz): δ (ppm) – 8.18 (d, 1H, J = 5.0 Hz, H_a); 7.83 (d, 1H, J = 8.6 Hz, H_d); 7.67 (t, 1H, J = 7.9 Hz, H_c); 7.62 (s, 1H, H_f); 7.10 (s, 2H, H_g, H_h); 6.87 (t, 1H, J = 5.5 Hz, H_b); 3.61 (s, $Me_{(e)}$).

Fig. S10. ^{13}C NMR spectra for ligand **L**[5] measured in CD_3OD.

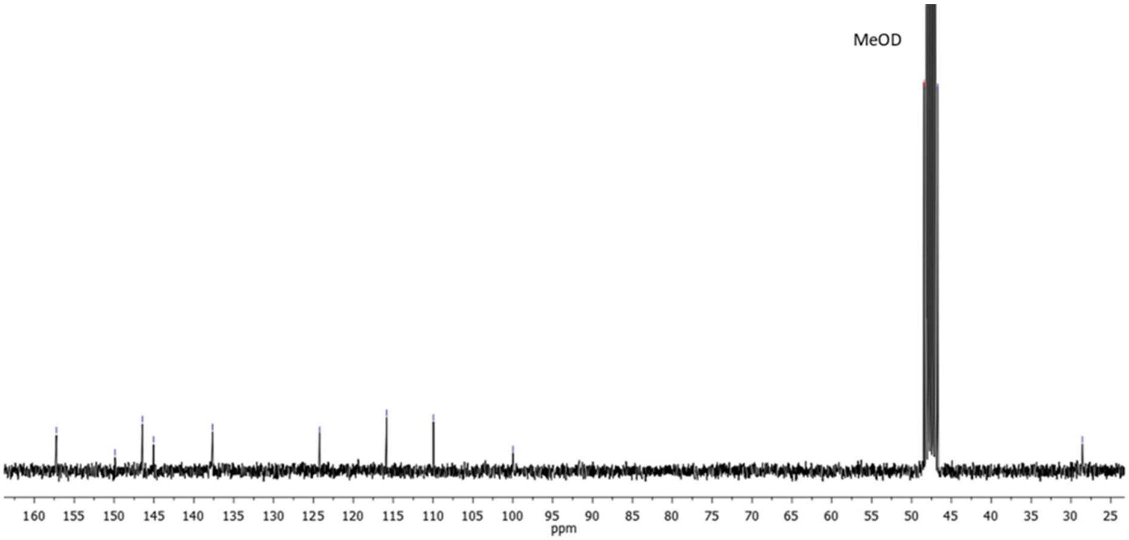

^{13}C NMR (CD_3OD, 75 MHz): δ (ppm) – 157.2, 149.9, 146.5, 145.1, 137.6, 124.2, 115.9, 109.9, 99.9, 28.6.

Fig. S11. ^{1}H NMR spectra for complex **10** measured in CD_3CN.

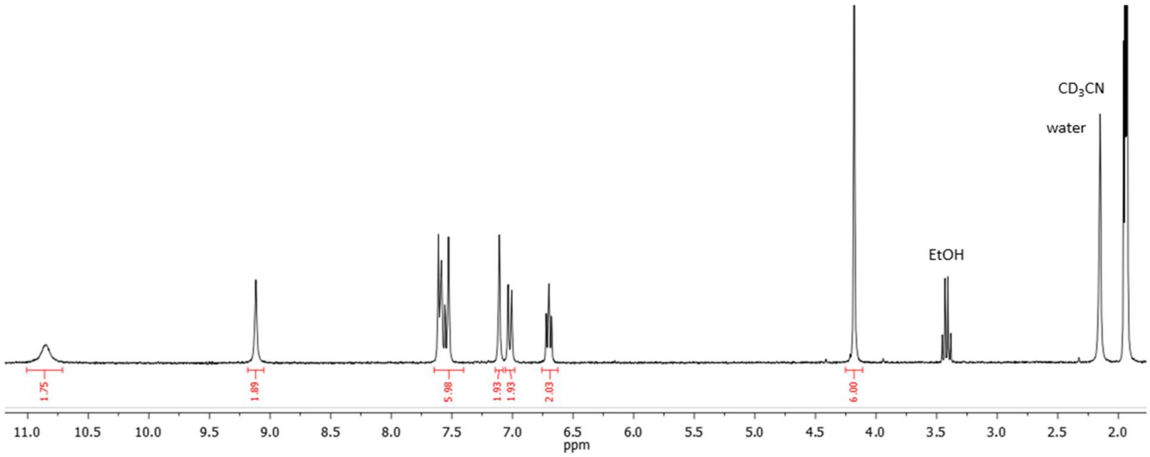

^{1}H NMR (CD_3CN, 300 MHz): δ (ppm) - 10.85 (s, 2H); 9.12 (s, 2H); 7.64-7.40 (m, 6H); 7.10 (s, 2H); 7.01 (d, 2H); 6.71 (t, 2H); 4.18 (s, 6H).

Fig. S12. ^{1}H NMR spectra for complex **11** measured in CD_3CN.

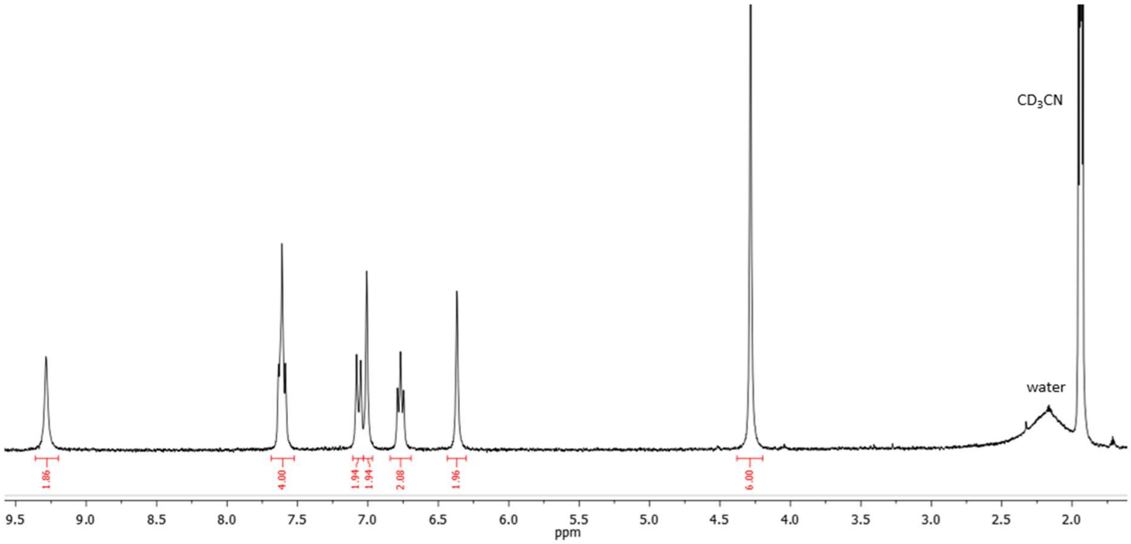

^{1}H NMR (CD_3CN, 300 MHz): δ (ppm) - 9.28 (s, 2H); 7.69-7.52 (m, 4H); 7.06 (d, 2H); 7.01 (s, 2H); 6.77 (t, 2H); 6.37 (s, 2H); 4.28 (s, 6H).

Fig. S13. ^{1}H NMR spectra for complex **12** measured in CD_3CN.

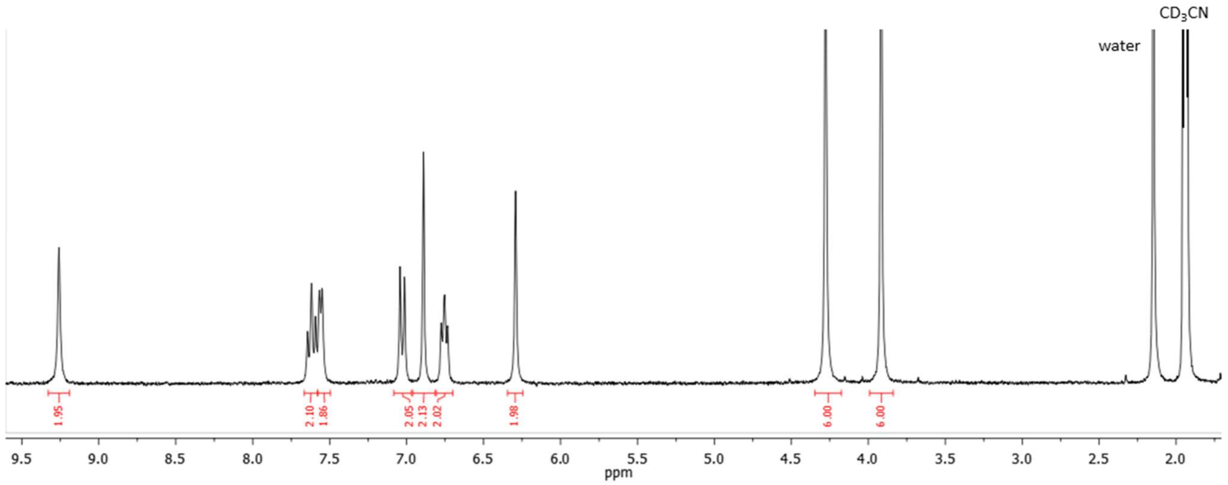

^{1}H NMR (CD_3CN, 300 MHz): δ (ppm) - 9.26 (s, 2H); 7.69 (t, 2H); 7.56 (d, 2H); 7.03 (d, 2H); 6.89 (s, 2H); 6.75 (t, 2H); 6.29 (s, 2H); 4.28 (s, 6H); 3.92 (s, 6H).

2. ESI-MS spectra of ligand and complexes

Fig. S14. ESI-MS spectra for ligand **L**[1].

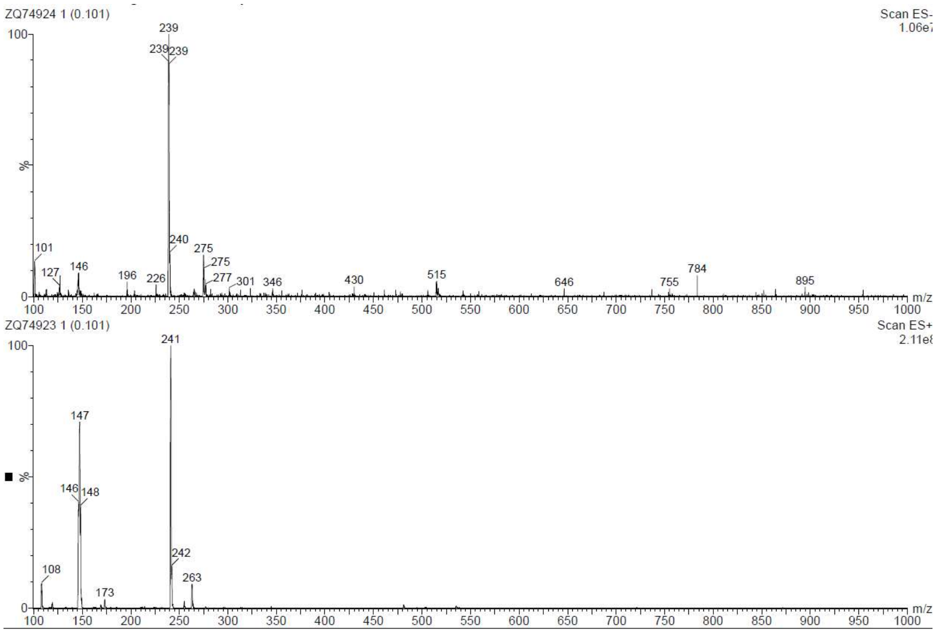

ESI-MS(+) m/z (%): 241 (100) [H**L**[1]]$^+$, 263 (10) [Na**L**[1]]$^+$; ESI-MS(-): 239 (100) [**L**[1]-H]$^-$.

Fig. S15. ESI-MS spectra for ligand **L**[2].

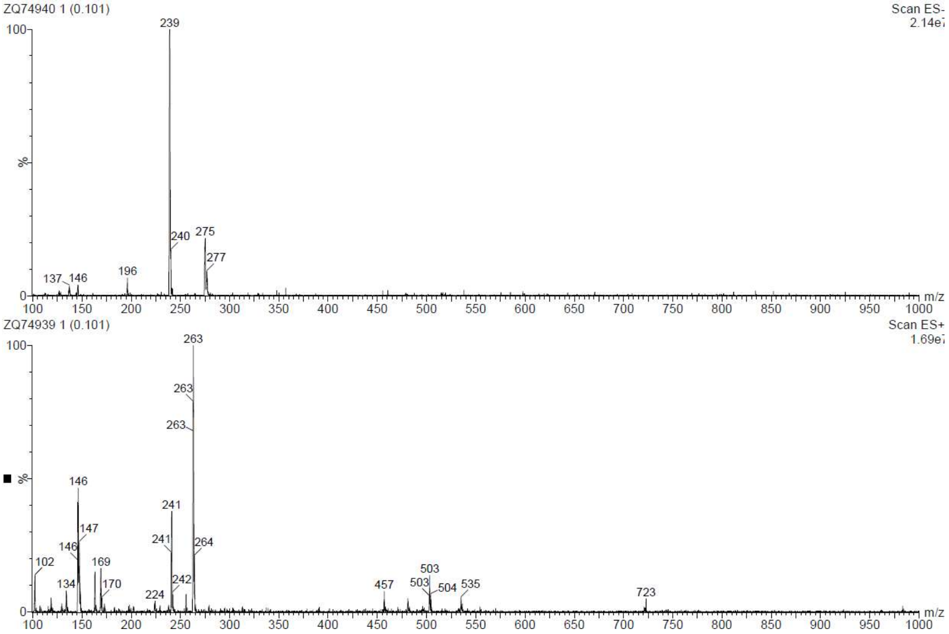

ESI-MS(+) m/z (%): 241 (40) [H**L**[2]]$^+$, 263 (100) [Na**L**[2]]$^+$; ESI-MS(-): 239 (100) [**L**[2]-H]$^-$.

Fig. S16. ESI-MS spectra for ligand **L**^{3}.

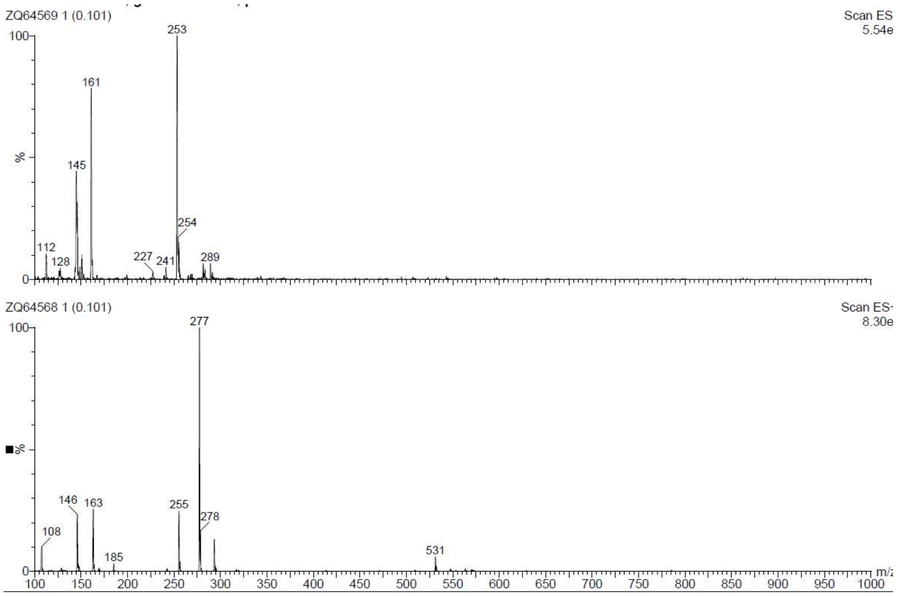

ESI-MS(+) m/z (%): 255 (30) [H**L**^{3}]$^+$, 277 (100) [Na**L**^{3}]$^+$; ESI-MS(-): 253 (100) [**L**^{3}-H]$^-$.

Fig. S17. ESI-MS spectra for ligand **L**^{4}.

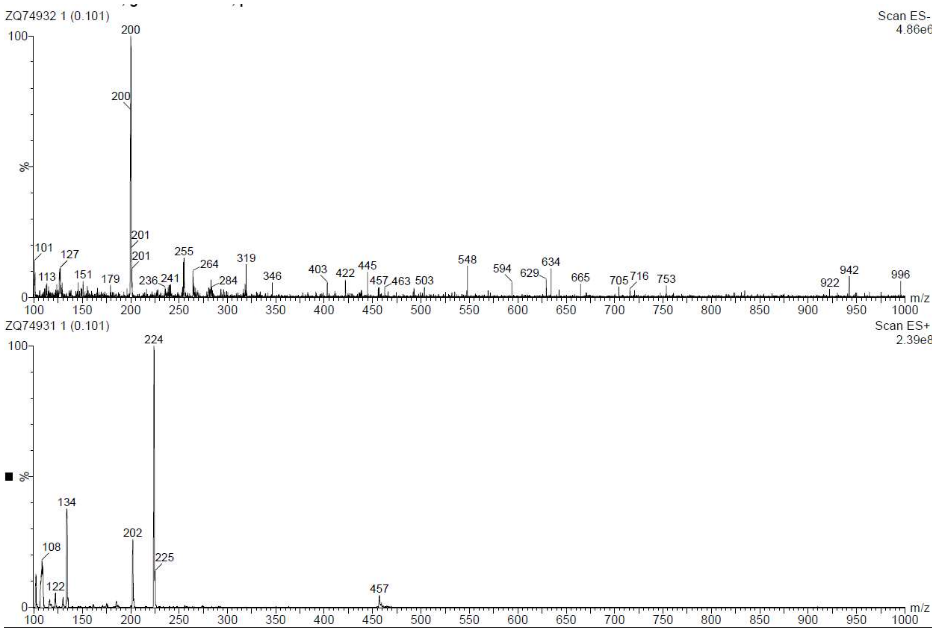

ESI-MS(+) m/z (%): 202 (20) [H**L**^{4}]$^+$, 224 (25) [Na**L**^{4}]$^+$; ESI-MS(-): 200 (100) [**L**^{4}-H]$^-$.

Fig. S18. ESI-MS spectra for ligand $\mathbf{L}^5$.

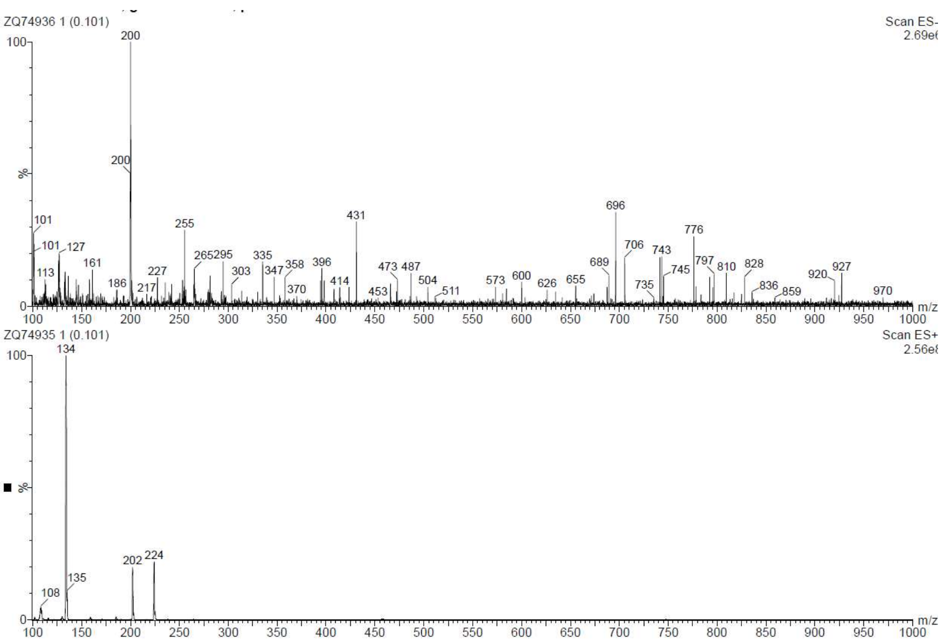

ESI-MS(+) m/z (%): 202 (30) [H$\mathbf{L}^5$]$^+$, 224 (100) [Na$\mathbf{L}^5$]$^+$; ESI-MS(-): 200 (100) [$\mathbf{L}^5$-H]$^-$.

Fig. S19. ESI-MS spectra for ligand $\mathbf{L}^6$.

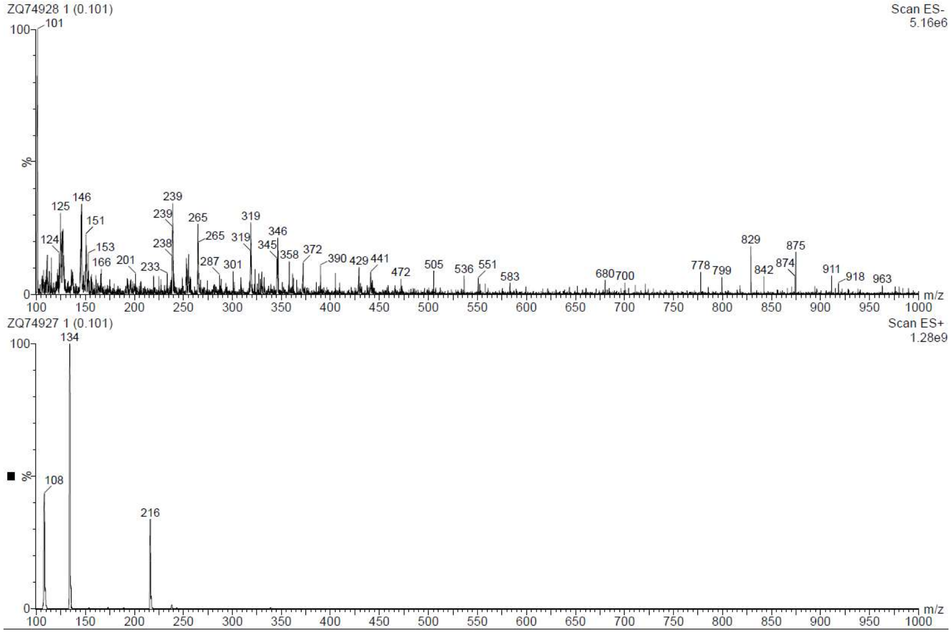

ESI-MS(+) m/z (%): 216 (100) [H$\mathbf{L}^6$]$^+$.

Fig. S20. ESI-MS spectra for complex **1**.

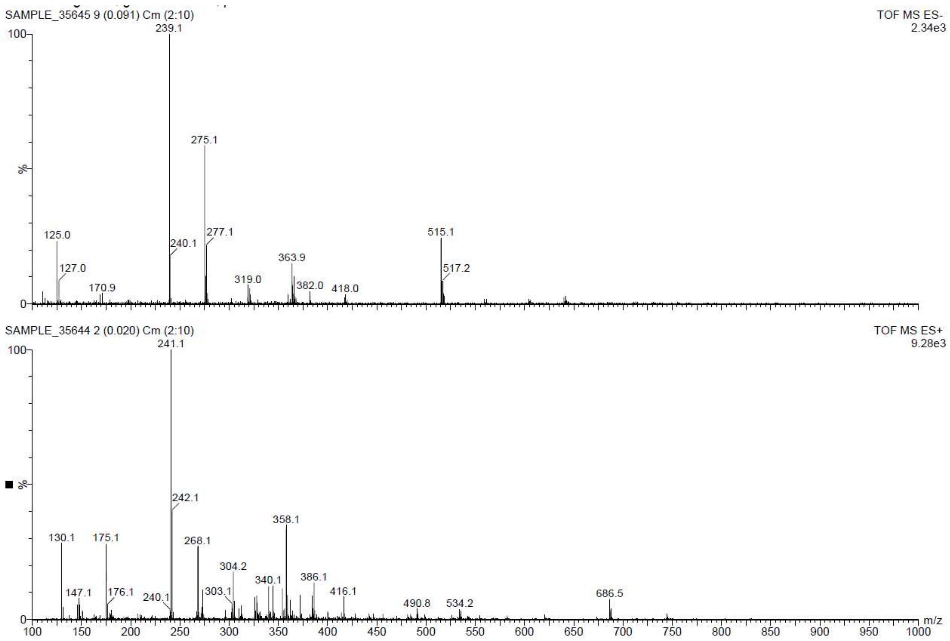

ESI-MS(+) m/z (%): 241 (100) [H**L**[1]]$^+$; ESI-MS(-) m/z (%): 239 (100) [**L**[1]-H]$^-$.

Fig. S21. ESI-MS spectra for complex **2**.

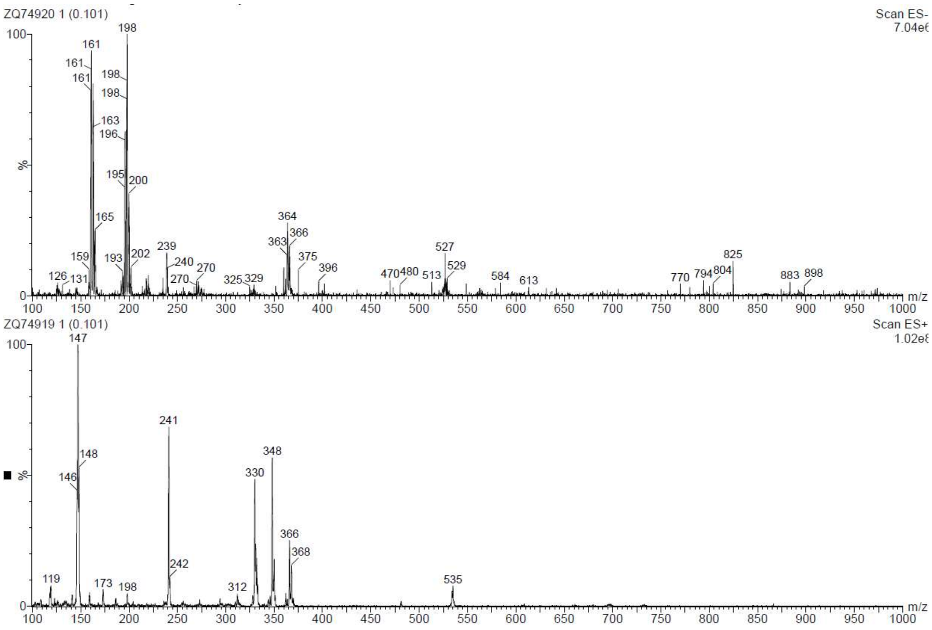

ESI-MS(+) m/z (%): 241 (70) [H**L**[2]]$^+$, 366 (30) [Fe**L**[2]Cl_2]$^+$; ESI-MS(-) m/z (%): 198 (100) [$FeCl_4$]$^-$.

Fig. S22. ESI-MS spectra for complex **3**.

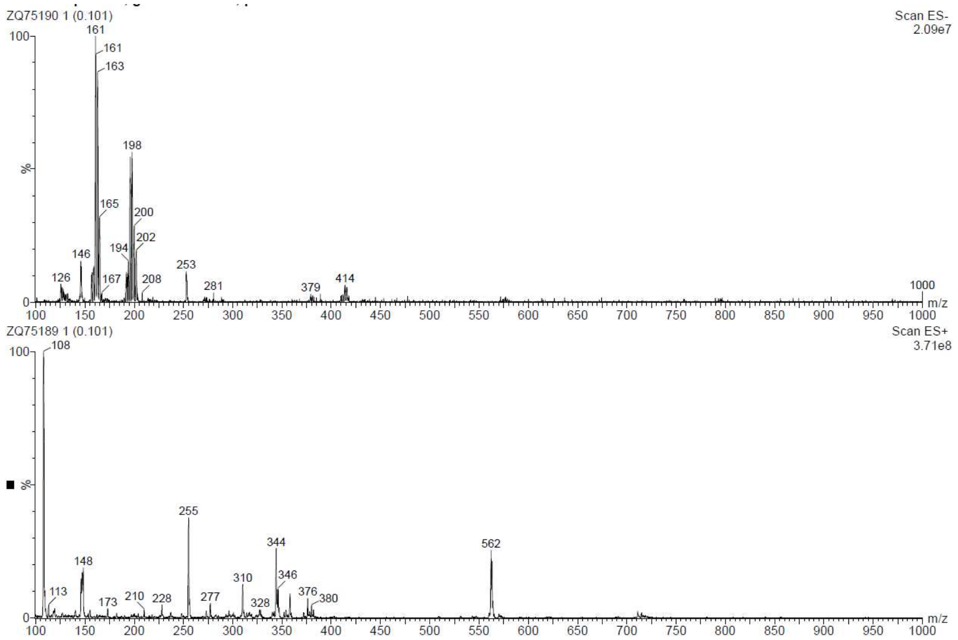

ESI-MS(+) m/z (%): 255 (40) [H**L**^{3}]$^+$, 277 (5) [Na**L**^{3}]$^+$, 344 (30) [Fe(**L**^{3}-H)Cl]$^+$.

Fig. S23. ESI-MS spectra for complex **4**.

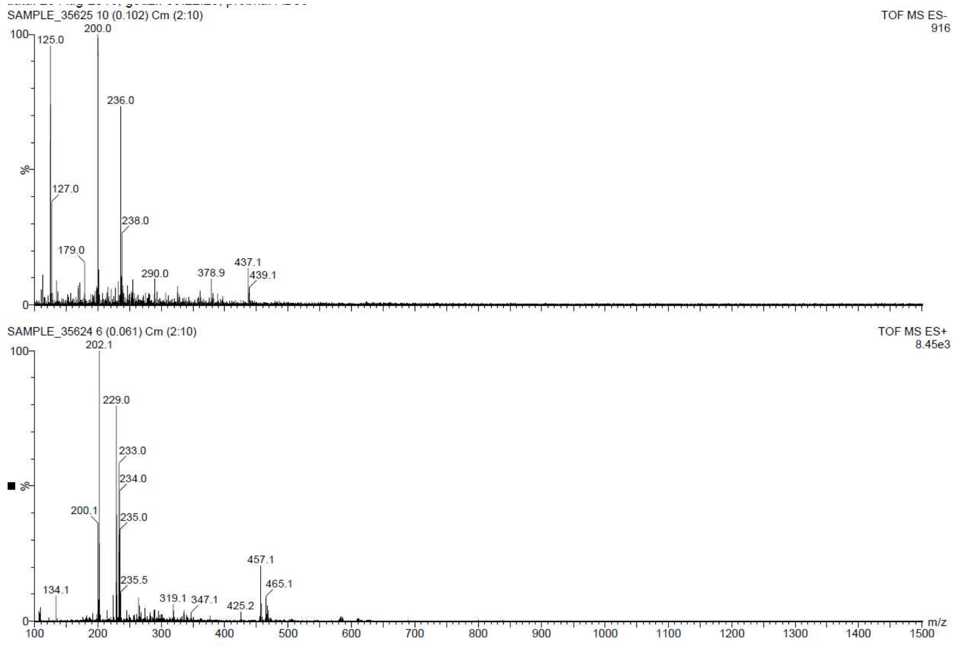

ESI-MS(+) m/z (%): 202 (100) [H**L**^{4}]$^+$; ESI-MS(-) m/z (%): 200 (100) [**L**^{4}-H]$^-$.

Fig. S24. ESI-MS spectra for complex **5**.

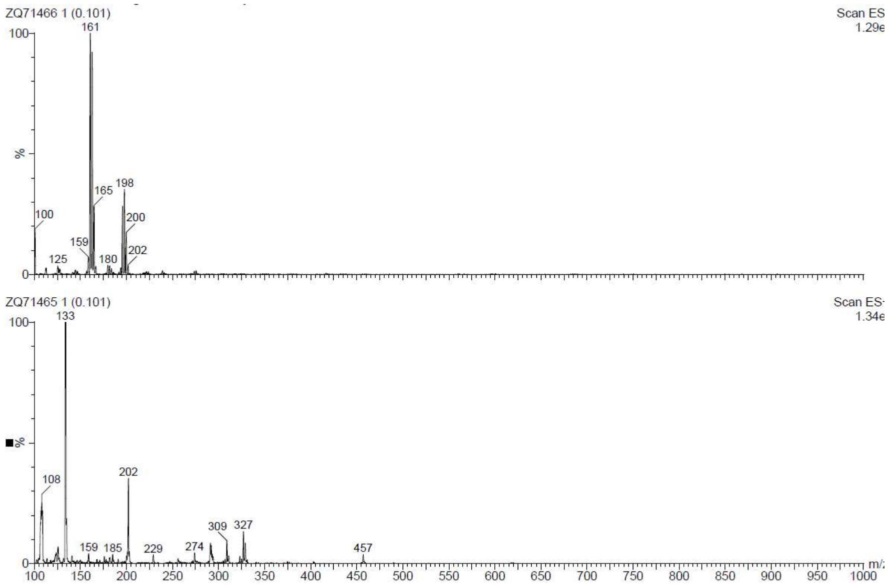

ESI-MS(+) m/z (%): 202 (40) $[H\mathbf{L}^5]^+$, 327 (20) $[Fe\mathbf{L}^5Cl_2]^+$; ESI-MS(-) m/z (%): 198 (40) $[FeCl_4]^-$.

Fig. S25. ESI-MS spectra for complex **6**.

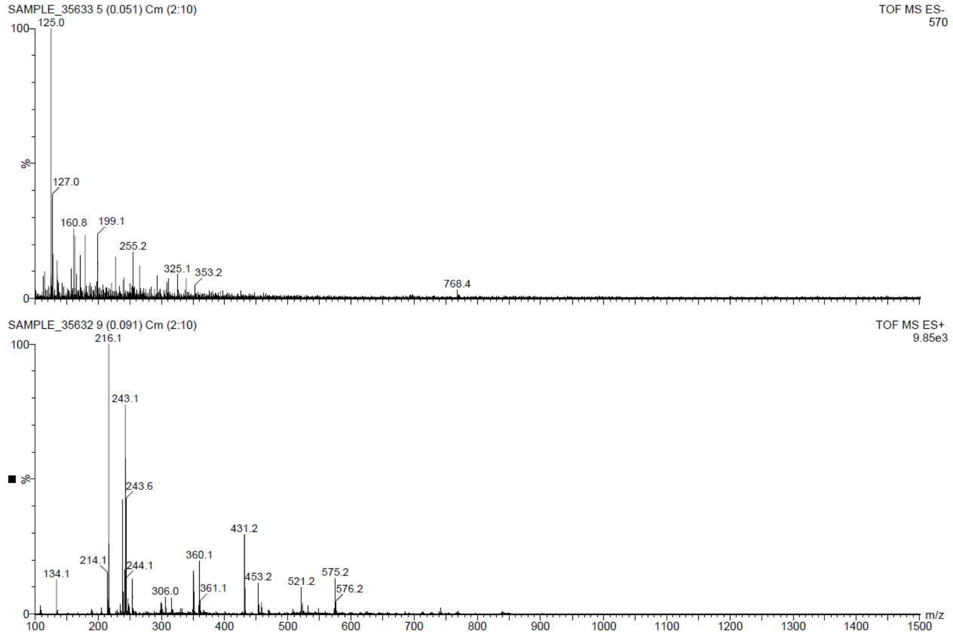

ESI-MS(+) m/z (%): 216 (100) $[H\mathbf{L}^6]^+$.

Fig. S26. ESI-MS spectra for complex **7**.

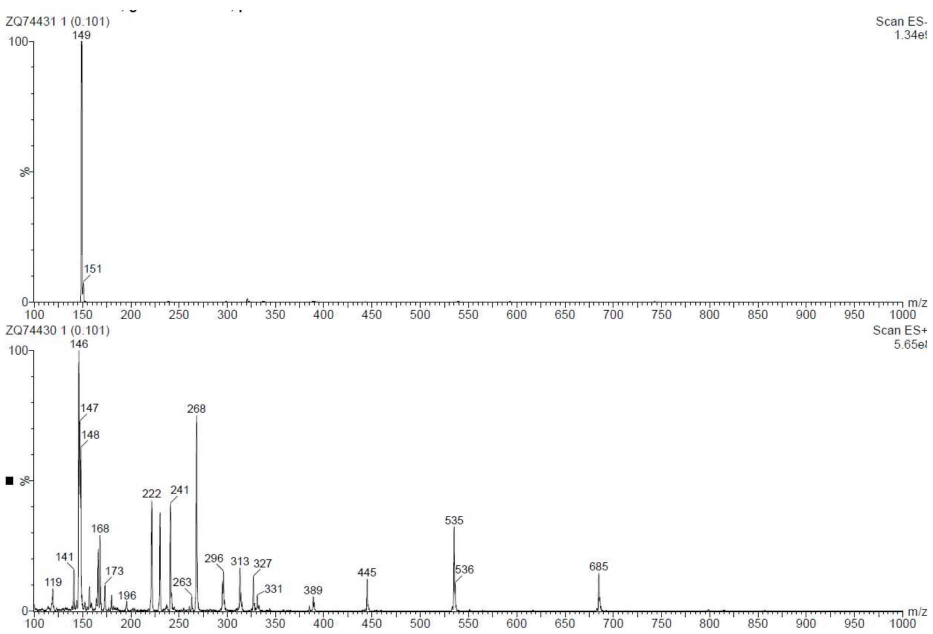

ESI-MS(+) m/z (%): 241 (40) [H**L**^{1}]$^+$, 263 (5) [Na**L**^{1}]$^+$, 268 (80) [Fe**L**$^1_2$]$^{2+}$, 535 (35) [Fe**L**^{1}(**L**^{1}-H)]$^+$; ESI-MS(-): 149 (100) [CF_3SO_3]$^-$.

Fig. S27. ESI-MS spectra for complex **8**.

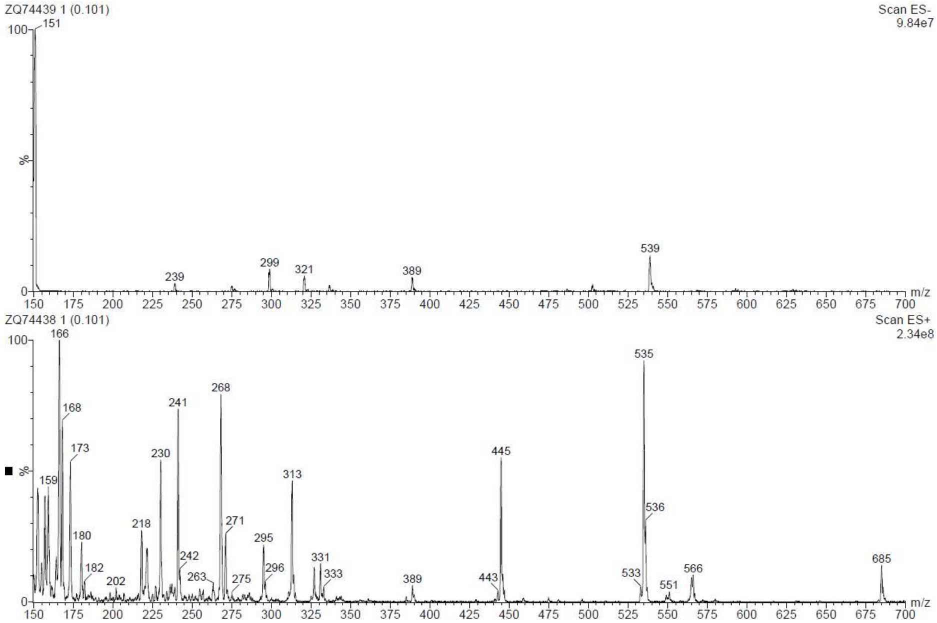

ESI-MS(+) m/z (%): 241 (75) [H**L**^{2}]$^+$**,** 263 (5) [Na**L**^{2}]$^+$, 268 (80) [Fe**L**$^2_2$]$^{2+}$, 535 (95) [Fe**L**^{2}(**L**^{2}-H)]$^+$.

Fig. S28. ESI-MS spectra for complex **9**.

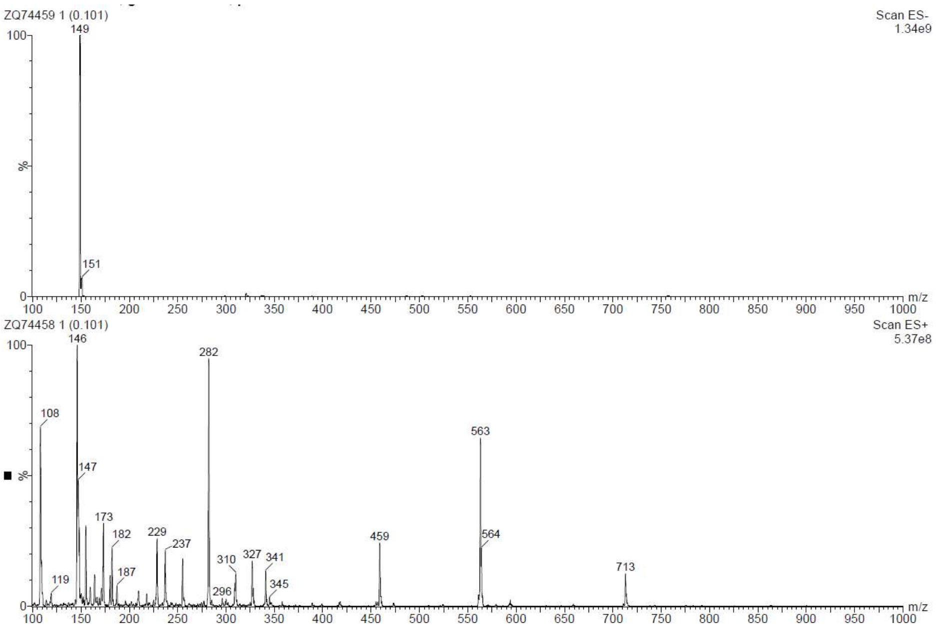

ESI-MS(+) m/z (%): 282 (95) [FeL^3_2]$^{2+}$, 563 (70) [FeL^3(L^3-H)]$^+$; ESI-MS(-): 149 (100) [CF_3SO_3]$^-$.

Fig. S29. ESI-MS spectra for complex **10**.

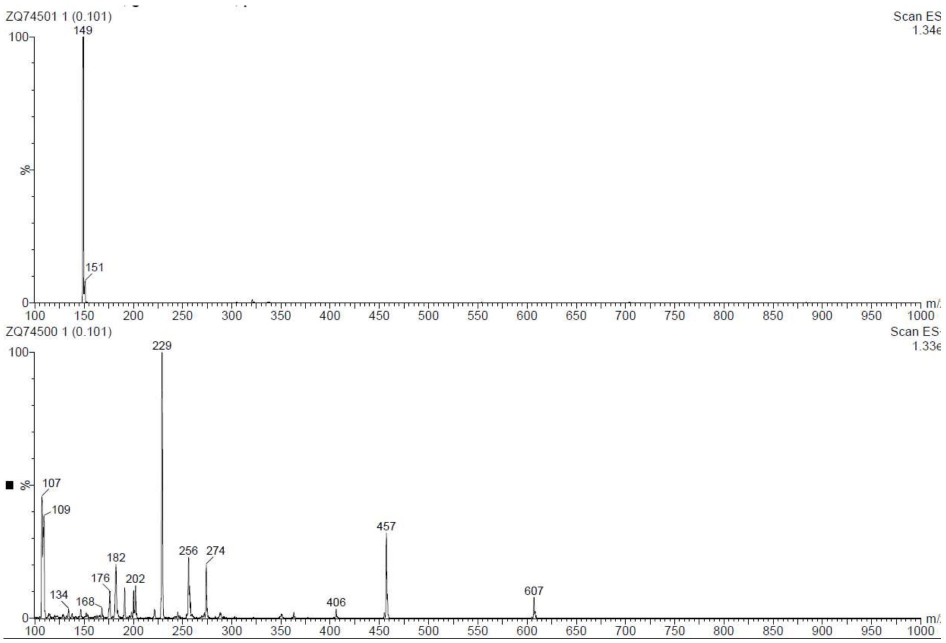

ESI-MS(+) m/z (%): 229 (100) [FeL^4_2]$^+$, 457 (35) [FeL^4(L^4-H)]$^+$; ESI-MS(-): 149 (100) [CF_3SO_3]$^-$.

Fig. S30. ESI-MS spectra for complex **11**.

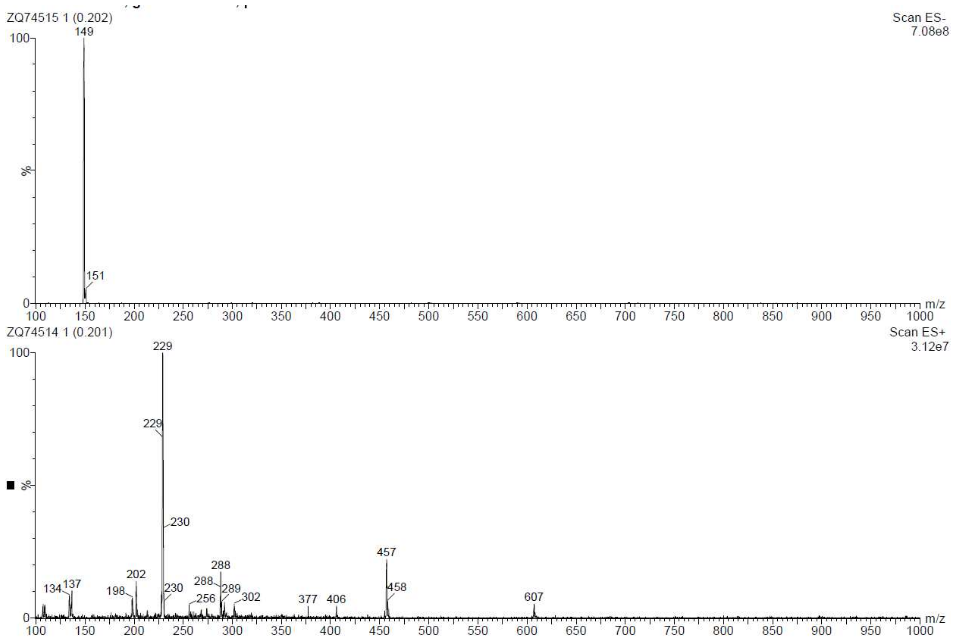

ESI-MS(+) m/z (%): 229 (100) $[Fe\mathbf{L}^{5}{}_{2}]^{2+}$, 457 (20) $[Fe\mathbf{L}^{5}(\mathbf{L}^{5}\text{-H})]^{+}$; ESI-MS(-): 149 (100) $[CF_3SO_3]^{-}$.

Fig. S31. ESI-MS spectra for complex **12**.

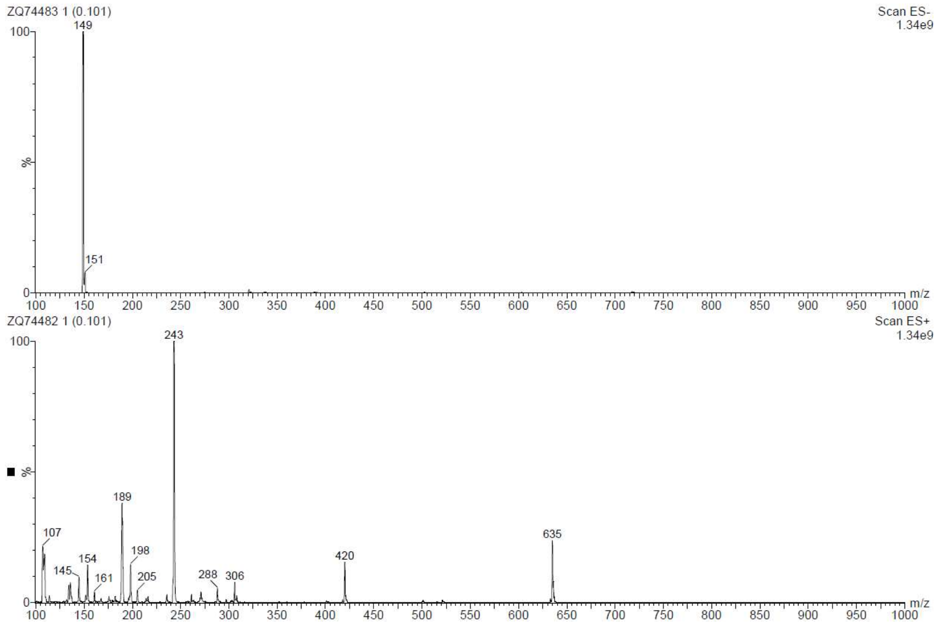

ESI-MS(+) m/z (%): 243 (100) $[Fe\mathbf{L}^{6}{}_{2}]^{2+}$, 635 (30) $[Fe\mathbf{L}^{6}{}_{2}(CF_3SO_3)]^{+}$; ESI-MS(-): 149 (100) $[CF_3SO_3]^{-}$.

3. Crystal data and structure of complexes

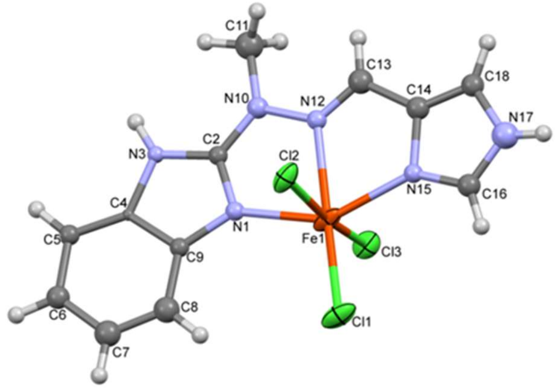

Fig. S32. Anisotropic-ellipsoid representation of the complex **1** the ellipsoids are drawn at the 50% probability level, hydrogen atoms are shown as spheres of arbitrary radii.

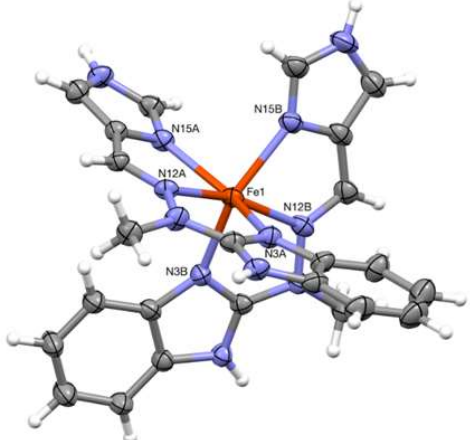

Fig. S33. Anisotropic-ellipsoid representation of the complex **7** the ellipsoids are drawn at the 50% probability level, hydrogen atoms are shown as spheres of arbitrary radii.

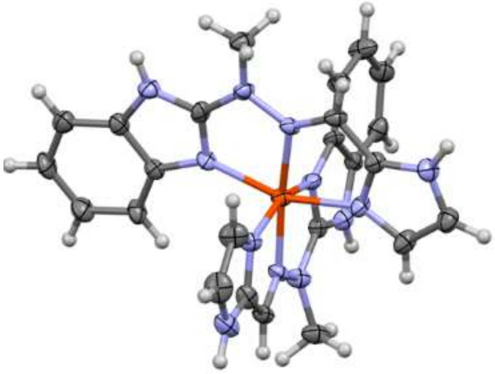

Fig. S34. Anisotropic-ellipsoid representation of the complex **8** the ellipsoids are drawn at the 50% probability level, hydrogen atoms are shown as spheres of arbitrary radii.

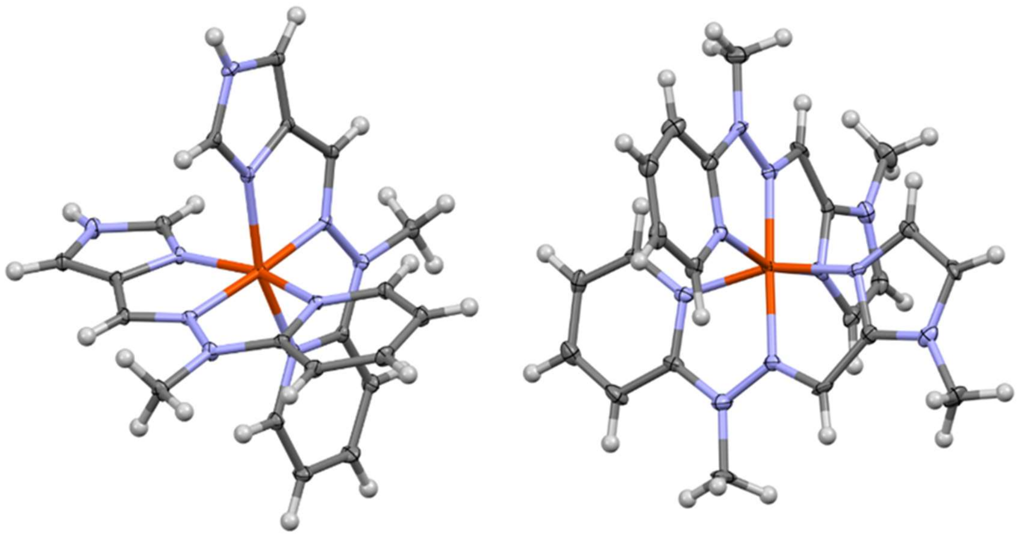

Fig. S35. Anisotropic-ellipsoid representation of the complex **10** and **12** the ellipsoids are drawn at the 50% probability level, hydrogen atoms are shown as spheres of arbitrary radii.

Table S1. Relevant geometrical data (Å, º); “angles” are three largest angles around Fe center, A, B and C denote mean planes of benzimidazole or pyridine ring, chain linker and imidazole ring, respectively.

	**1**	**2**	**4**	**6**
Fe1-N3 (N1)	2.043(15)	2.0736(19)	2.137(5)	2.1161(18)
Fe1-N12	2.234(15)	2.226(2)	2.172(5)	2.1911(17)
Fe1-N15	2.090(15)	2.096(2)	2.087(5)	2.0786(18)
Fe1-Cl	2.234(6)<br>2.318(6)<br>2.543(6)	2.2518(7)<br>2.2883(7)	2.2538(17)<br>2.3045(17)<br>2.4771(17)	2.2584(6)<br>2.3365(6)<br>2.3890(6)
Fe1-O		2.1217(17)		
angles	174.7(4)<br>173.0(2)<br>147.1(6)	169.43(5)<br>169.00(6)<br>146.11(8)	173.34(7)<br>172.20(15)<br>147.2(2)	178.07(5)<br>171.30(2)<br>147.17(7)
A/B	7.1(12)	2.14(13)	7.3(3)	6.52(9)

B/C	6.9(15)	4.50(16)	8.3(4)	3.05(10)
A/C	10.7(9)	3.80(14)	10.4(4)	6.31(12)

	**7**	**8**	**9**	**10**	**12**
Fe1-N3 (N1)	2.152(3)<br>2.140(3)	1.987(5)<br>2.003(5)	2.164(2)<br>2.132(2)	1.9571(14)	1.941(7)<br>1.956(7)
Fe1-N12	2.184(3)<br>2.183(3)	1.957(4)<br>1.976(4)	2.173(3)<br>2.199(3)	1.9521(14)	1.888(6)<br>1.891(7)
Fe1-N15	2.191(3)<br>2.173(3)	1.997(5)<br>2.008(5)	2.207(3)<br>2.132(2)	1.9014(13)	1.984(7)<br>1.996(7)
angles	166.00(10)<br>145.85(11)<br>143.14(11)	171.91(18)<br>158.22(19)<br>158.13(19)	163.79(9)<br>146.17(9)<br>146.09(9)	1.8988(13)	177.5(3)<br>162.1(3)<br>161.9(3)
				1.9715(14)	
A/B	9.55(15)<br>10.20(14)	6.7(3)<br>3.3(3)	14.05(7)<br>6.67(9)	1.9558(14)	1.9(3)<br>3.4(3)
B/C	14.34(17)<br>8.8(2)	8.2(4)<br>3.5(4)	5.97(12)<br>7.02(14)	176.05(6)	1.1(3)<br>5.3(3)
A/C	15.90(17)<br>18.69(18)	6.1(4)<br>3.1(4)	17.88(8)<br>13.55(12)	161.93(6)	1.9(4)<br>7.8(4)
A/A′	86.41(7)	83.36(10)	74.65(6)	161.82(6)	88.3(2)
Voids	17.0	12.4	14.4		5.8

Table S2. Hydrogen bond data (Å, º)

D	H	A	D-H	H···A	D···A	D-H···A
**1**						
N3	H3	$Cl2^{i}$	0.88	2.37	3.145(16)	147
N17	H17	$Cl2^{ii}$	0.88	2.41	3.203(18)	151
**2**						

N3	H3	Cl3^{i}	0.88	2.21	3.083(2)	174
N18	H18	Cl3^{iii}	0.88	2.24	3.051(2)	154
O19	H19	Cl3	0.86	2.10	2.9431(18)	171
**4**						
N14	H14	Cl3^{iv}	0.88	2.44	3.179(6)	142
**7**						
N1A	H1A	O2C^{vi}	0.88	2.04	2.906(4)	166
N17A	H17A	O3C	0.88	1.93	2.812(4)	175
N1B	H1B	O3D	0.88	2.02	2.895(4)	173
N17B	H17B	O2D^{vii}	0.88	2.05	2.914(4)	165
**8**						
N1A	H1A	O2D^{viii}	0.88	2.16	2.904(6)	142
N18A	H18A	O3C	0.88	2.26	2.856(7)	125
N18A	H18A	O3D	0.88	2.28	3.007(6)	140
N1B	H1B	O1D^{ix}	0.88	1.97	2.833(6)	166
N18B	H18B	O3D^{x}	0.88	2.09	2.961(6)	173
**9**						
N1A	H1A	O3C^{xi}	0.88	1.97	2.814(3)	161
N1B	H1B	O3D^{xii}	0.88	2.03	2.896(3)	166
**10**						
N14A	H14A	O2D	0.88	1.98	2.862(7)	179
N14B	H14B	O3C	0.88	1.95	2.811(7)	165
**12**						
C6A	H6A	O1E	0.95	2.35	3.196(12)	148
C10C	H10C	O3H^{xiii}	0.95	2.23	3.158(11)	167
C6D	H6D	F2F	0.95	2.23	2.937(13)	130

Symmetrycodes: ^{i} 1-x,-1-y,1-z; ^{ii} 1-x,-y,2-z; ^{iii} 3/2-x,1/2+y,1/2-z; ^{iv} -x,1-y,1-z; ^{v} -1+x,y,z; ^{vi} 1/2+x,1/2+y, z; ^{vii} -1/2+x,1/2+y,z; ^{viii} 1–x,1/2+y,3/2-z; ^{ix}x,1+y,z; ^{x} -x,1/2+y,3/2-z; ^{xi} -1+x,y,z; ^{xii}; ^{xiii} 2-x,1-y,1-z.

Table S3. Comparative table of ligands structure and features with some of their kinetic parameters.

Ligand	'Open' [Fe(**L**^{x})Cl$_3$] K$_M$[10^{-3}M) / TON [h^{-1}]	'Closed' [Fe(**L**^{x})$_2$](OTf)$_2$ K$_M$[10^{-3}M) / TON [h^{-1}]	Number of H bonds in:			Disposition of H bonds
			Ligand	'Open' complex	'Closed' complex	
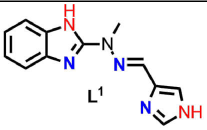 L1	1.45 / 185.25	3.98 / 199.40	2	2	4	a/e
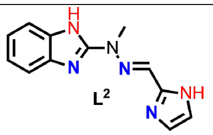 L2	2.16 / 127.30	1.96 / 97.68	2	2	4	a/a

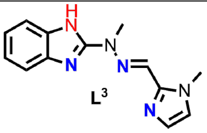 L^3	1.95 / 172.30	3.68 / 183.80	1	1	2	a/-
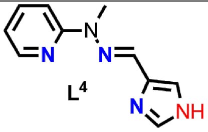 L^4	2.02 / 134.05	0.86 / 42.76	1	1	2	-/e
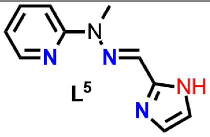 L^5	2.02 / 103.34	1.30 / 65.14	1	1	2	-/a
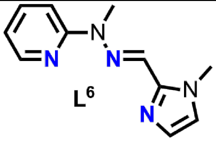 L^6	2.19 / 150.49	2.13 / 106.53	0	0	0	-/-

4. Spectra of the oxidation reactions

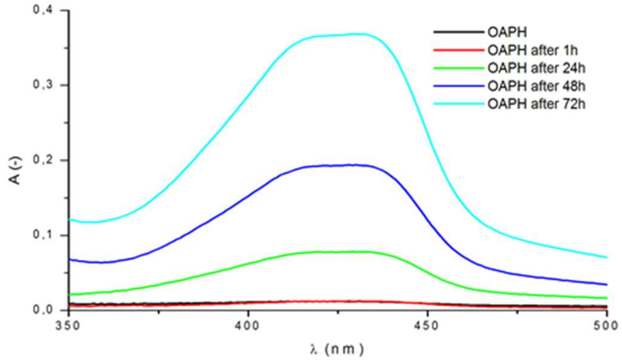

Fig. S36. The spectral profile showing blank test with 2-aminophenol dissolved in methanol. The spectra were recorded under aerobic conditions during three days. At 433 nm there is growth of 2-aminophenoxazine-3-one.

The spectra of the oxidation reaction of 2-aminophenol using complexes with a "open" system as catalysts:

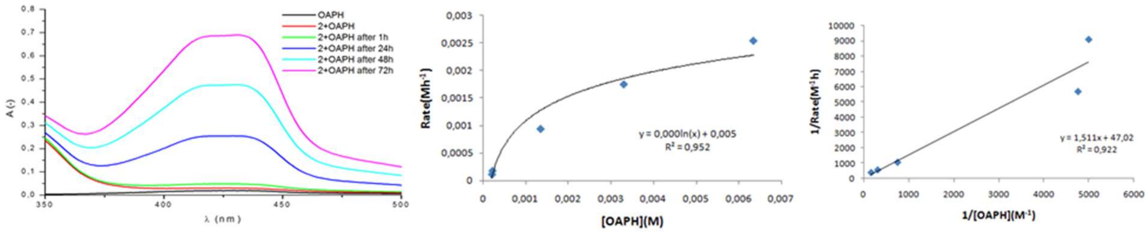

Fig. S37. The spectral profile showing the growth of 2-aminophenoxazine-3-one at 433 nm due to addition of complex **2** to 2-aminophenol dissolved in methanol. The spectra were recorded under aerobic conditions during three days (left). Plot of rate vs concentration for complex **2** (middle). Linewear-Burk plot of phenoxazinone synthase like activity for complex **2** (right).

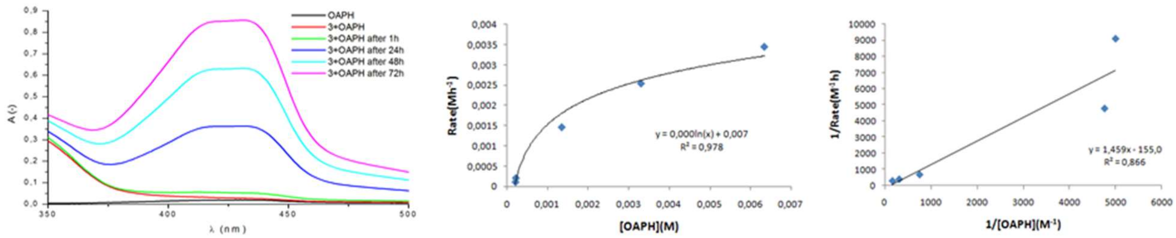

Fig. S38. The spectral profile showing the growth of 2-aminophenoxazine-3-one at 433 nm due to addition of complex **3** to 2-aminophenol dissolved in methanol. The spectra were recorded under aerobic conditions during three days (left). Plot of rate vs concentration for complex **3** (middle). Linewear-Burk plot of phenoxazinone synthase like activity for complex **3** (right).

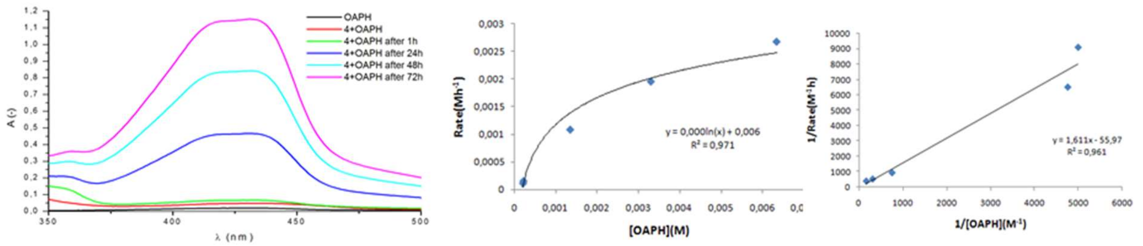

Fig. S39. The spectral profile showing the growth of 2-aminophenoxazine-3-one at 433 nm due to addition of complex **4** to 2-aminophenol dissolved in methanol. The spectra were recorded under aerobic conditions during three days (left). Plot of rate vs concentration for complex **4** (middle). Linewear-Burk plot of phenoxazinone synthase like activity for complex **4** (right).

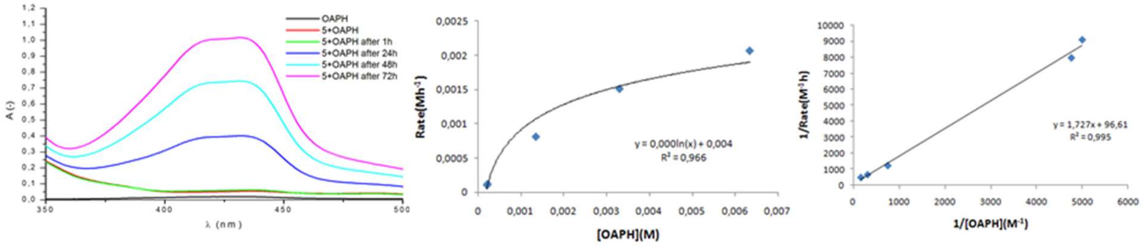

Fig. S40. The spectral profile showing the growth of 2-aminophenoxazine-3-one at 433 nm due to addition of complex **5** to 2-aminophenol dissolved in methanol. The spectra were recorded under aerobic conditions during three days (left). Plot of rate vs concentration for complex **5** (middle). Linewear-Burk plot of phenoxazinone synthase like activity for complex **5** (right).

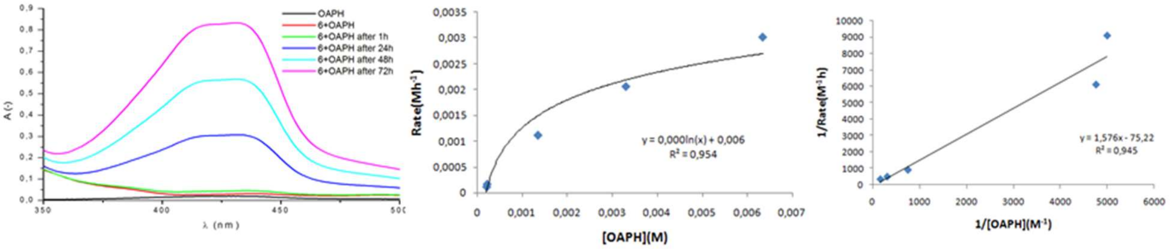

Fig. S41. The spectral profile showing the growth of 2-aminophenoxazine-3-one at 433 nm due to addition of complex **6** to 2-aminophenol dissolved in methanol. The spectra were recorded under aerobic conditions during three days (left). Plot of rate vs concentration for complex **6** (middle). Linewear-Burk plot of phenoxazinone synthase like activity for complex **6** (right).

The spectra of the oxidation reaction of 2-aminophenol using complexes with a “closed” system as catalysts:

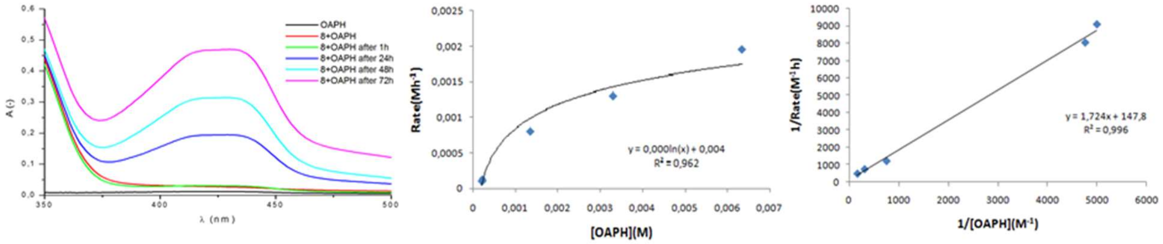

Fig. S42. The spectral profile showing the growth of 2-aminophenoxazine-3-one at 433 nm due to addition of complex **8** to 2-aminophenol dissolved in methanol. The spectra were recorded under aerobic conditions during three days (left). Plot of rate vs concentration for complex **8** (middle). Linewear-Burk plot of phenoxazinone synthase like activity for complex **8** (right).

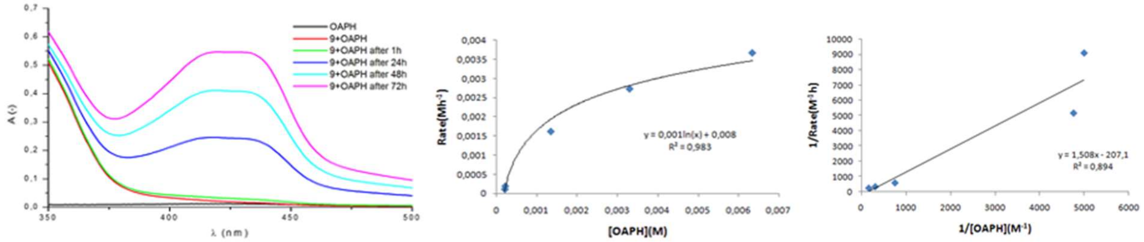

Fig. S43. The spectral profile showing the growth of 2-aminophenoxazine-3-one at 433 nm due to addition of complex **9** to 2-aminophenol dissolved in methanol. The spectra were recorded under aerobic conditions during three days (left). Plot of rate vs concentration for complex **9** (middle). Linewear-Burk plot of phenoxazinone synthase like activity for complex **9** (right).

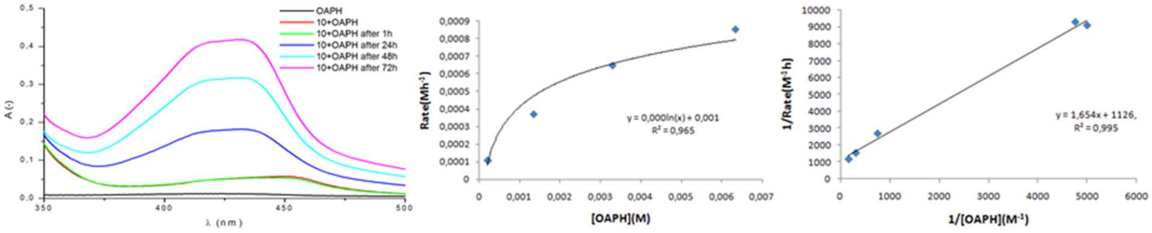

Fig. S44. The spectral profile showing the growth of 2-aminophenoxazine-3-one at 433 nm due to addition of complex **10** to 2-aminophenol dissolved in methanol. The spectra were recorded under aerobic conditions during three days (left). Plot of rate vs concentration for complex **10** (middle). Linewear-Burk plot of phenoxazinone synthase like activity for complex **10** (right).

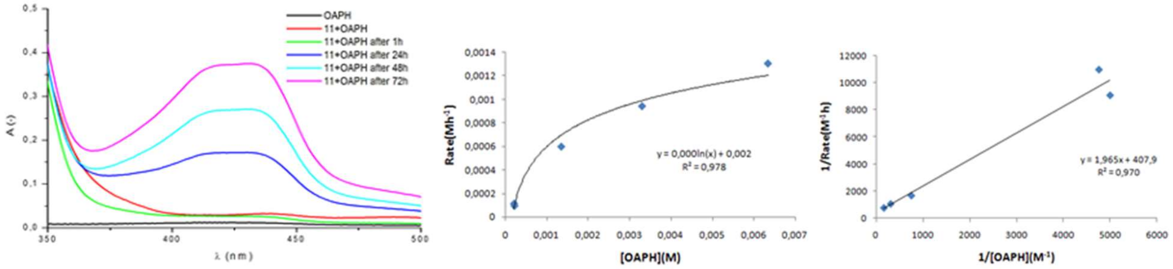

Fig. S45. The spectral profile showing the growth of 2-aminophenoxazine-3-one at 433 nm due to addition of complex **11** to 2-aminophenol dissolved in methanol. The spectra were recorded under aerobic conditions during three days (left). Plot of rate vs concentration for complex **11** (middle). Linewear-Burk plot of phenoxazinone synthase like activity for complex **11** (right).

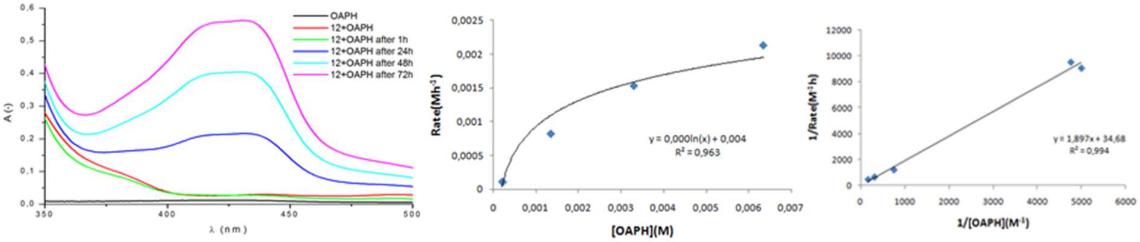

Fig. S46. The spectral profile showing the growth of 2-aminophenoxazine-3-one at 433 nm due to addition of complex **12** to 2-aminophenol dissolved in methanol. The spectra were recorded under aerobic conditions during three days (left). Plot of rate vs concentration for complex **12** (middle). Linewear-Burk plot of phenoxazinone synthase like activity for complex **12** (right).

5. Biological spectra

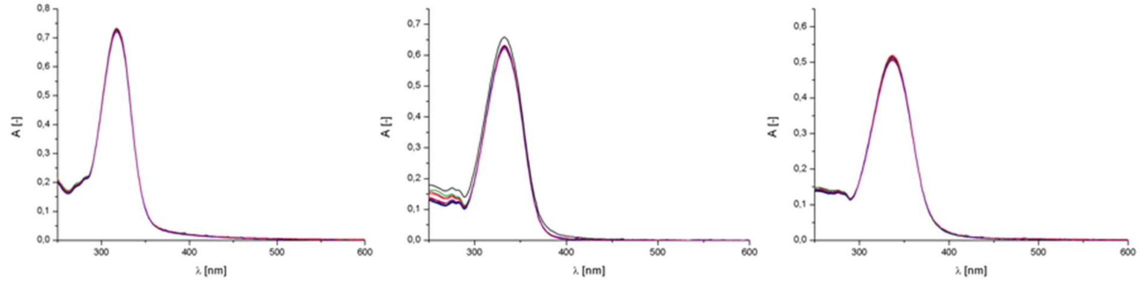

Fig. S47. Absorption titration of **1** (left)**,** **2** (middle) and **3** (right) with increasing concentrations of CT-DNA (0-100 μM).

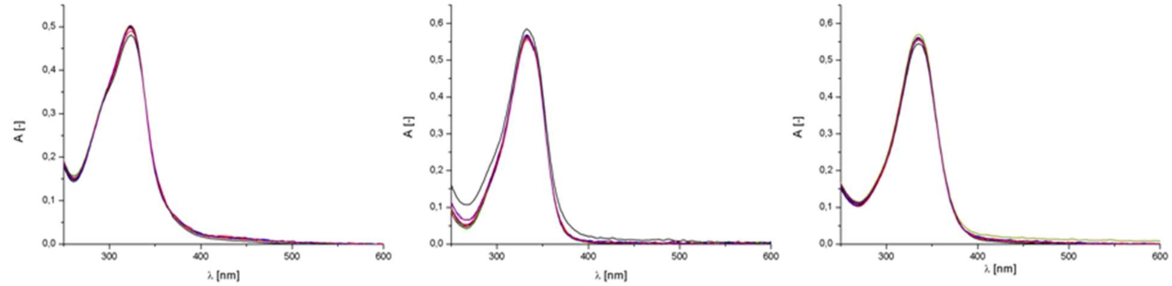

Fig. S48. Absorption titration of **4** (left)**,** **5** (middle) and **6** (right) with increasing concentrations of CT-DNA (0-100 μM).

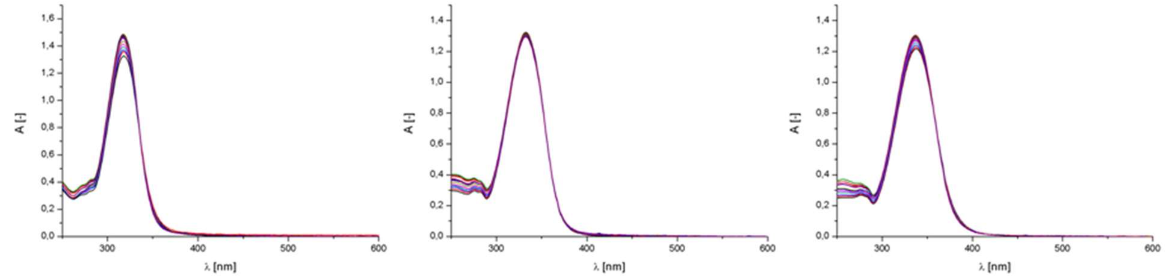

Fig. S49. Absorption titration of **7** (left), **8** (middle) and **9** (right) with increasing concentrations of CT-DNA (0-100 μM).

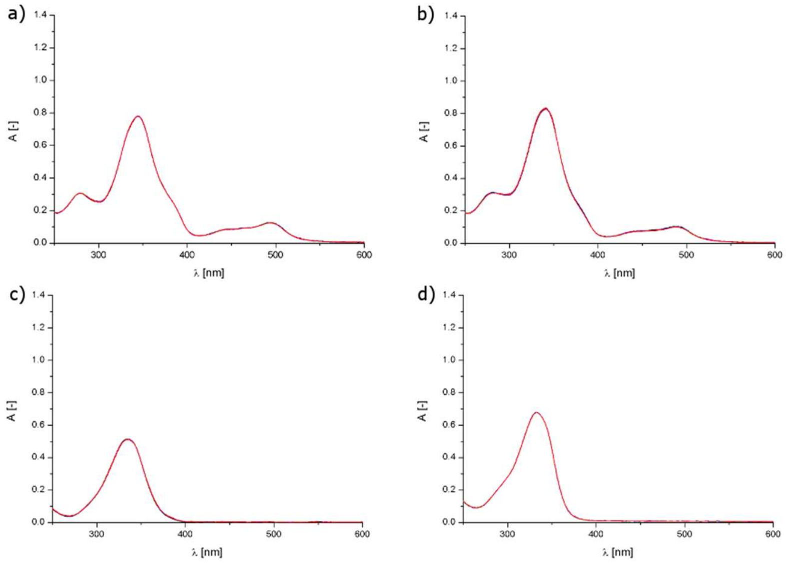

Fig. S50. Stability test of complex compounds **12** (a), **11** (b) and ligands $\mathbf{L^6}$ (c), $\mathbf{L^5}$ (d) over time (0-420min).

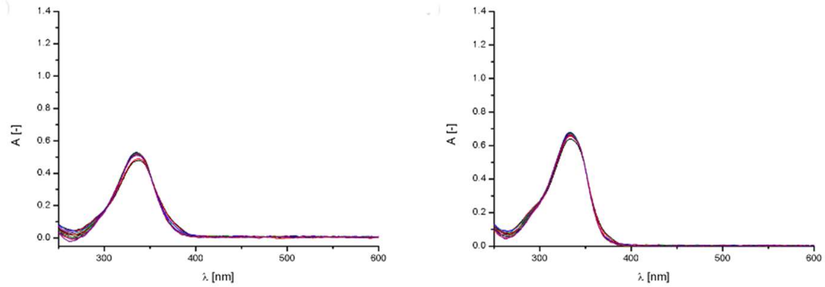

Fig. S51 Absorption titration of $\mathbf{L^6}$ (left) and $\mathbf{L^5}$ (right) with increasing concentrations of CT-DNA (0-100 μM).

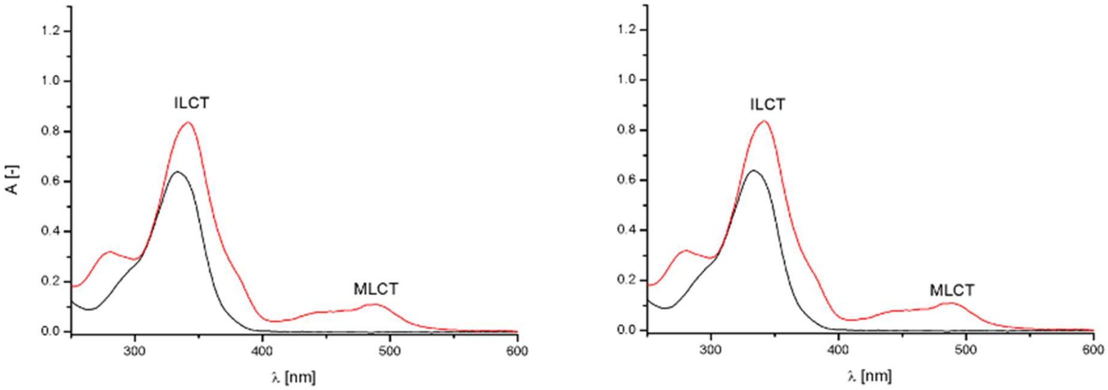

Fig. S52 Absorption spectra of $\mathbf{L^6}$ and **12** (left), $\mathbf{L^5}$ and **11** (right) in Tris-HCl buffer (pH=7.4).
